# Efficient synthesis of 1,3-diaryl-4-halo-1*H*-pyrazoles from 3-arylsydnones and 2-aryl-1,1-dihalo-1-alkenes

**DOI:** 10.3762/bjoc.7.195

**Published:** 2011-12-12

**Authors:** Yiwen Yang, Chunxiang Kuang, Hui Jin, Qing Yang, Zhongkui Zhang

**Affiliations:** 1 Department of Chemistry, Tongji University, Siping Road 1239, Shanghai 200092, China; 2 College of Biological, Chemical Sciences and Engineering, Jiaxing University, Jiaxing 314001, China; 3 Department of Biochemistry, School of Life Sciences, Fudan University, Handan Road 220, Shanghai 200433, China

**Keywords:** C–H bond activation, cycloaddition, dihaloalkenes, pyrazole, sydnones

## Abstract

An efficient synthesis of 1,3-diaryl-4-halo-1*H*-pyrazoles was achieved. The synthesis involves the [3 + 2] dipolar cycloaddition of 3-arylsydnones and 2-aryl-1,1-dihalo-1-alkenes. The process proceeds smoothly in moderate to excellent yields. 1,3-Diaryl-4-halo-1*H*-pyrazoles are found to be important intermediates that can easily be converted into 1,2,5-triaryl-substituted pyrazoles via Pd-catalyzed C–H bond activation.

## Introduction

Over the past decade, pyrazoles as key motifs in biologically active compounds have received increasing attention from the synthetic community. Diazoles can be employed as a central building block in the synthesis of compound libraries in the pharmaceutical [1] and agrochemical [2] industries. Pyrazole and its derivatives are an important class of heterocyclic compounds. As medicines, many of them display anti-inflammatory [3], antimicrobial [3], antiplatelet [4], antiallergenic [5], antifungal [6], MAP Kinase inhibitor [7], and anticancer activities [8]. As pesticides, they are used as insecticides [9] and fungicides [10], and as well as antiviral [11] and antibacterial agents [12]. Pyrazoles are gaining interest as ligands for transition metals, and in the field of materials chemistry [13–14].

Pyrazole and its derivatives can be synthesized by several methods [15]. The most common approach is based on the condensation of hydrazines with 1,3-dicarbonyl compounds or their equivalents. However, the 1,3-dipolar cycloaddition offers a more convenient synthetic route. Sydnones are easily accessible aromatic compounds and versatile synthetic intermediates. They can be used as unusual, alternative cycloaddition substrates for pyrazole synthesis [16–17]. These dipolar compounds are readily prepared in two steps from N-functionalized amino acids, and are readily stored and handled. Methods have been disclosed for the [3 + 2] dipolar cycloaddition of sydnones with alkenyl silanes [18] and stannanes [18], alkenyl arenes [19], 1,3-dienes [20–21], α,β-unsaturated esters [19,22] and nitriles [23], phosphane oxides [24] or with alkynyl silanes [18], stannanes [18,25–26], arenes [27–28], esters [29–33], boronic esters [34–35]. However, the cycloaddition of sydnones with 1,1-dihaloalkenes is unknown, as is the direct formation of 4-halopyrazoles through the [3 + 2] dipolar cycloaddition of sydnones.

**Scheme 1:**
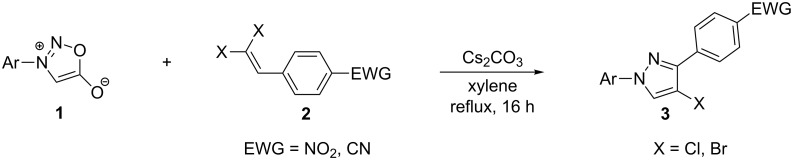
Access to 1,3-diaryl-4-halo-1*H*-pyrazoles from 3-arylsydnones and 2-aryl-1,1-dihalo-1-alkenes.

In the present study, a convenient and efficient synthesis of a series of new 1,3-diaryl-4-halo-1*H*-pyrazoles **3** in moderate to excellent yields is reported. The route employed involves 1,3-dipolar cycloaddition between 3-arylsydnones **1** and 2-aryl-1,1-dihalo-1-alkenes **2** (Scheme 1). 1,3-Diaryl-4-halo-1*H*-pyrazoles were found to be important intermediates that could easily be converted into 1,2,4-triaryl- or 1,2,5-triaryl-substituted pyrazoles via a Pd-catalyzed C–C coupling reaction. To the best of our knowledge, this synthesis of 1,3-diaryl-4-halo-1*H*-pyrazoles has not yet been reported.

## Results and Discussion

To determine the optimal reaction conditions, 3-phenylsydnone (**1a**) and 1-(2,2-dibromovinyl)-4-nitrobenzene (**2a**) were used as the model substrates. A mixture of **1a**, **2a**, Cs_2_CO_3_ and xylene was then stirred in the dark in a sealed tube maintained at 140 °C in an oil bath. After 16 h, the product **3a** was isolated in 72% yield.

The effects of different bases, molar ratios of **1a** to **2a**, solvents, and temperatures on the formation of **3a** were investigated. The optimization of the 1,3-dipolar cycloaddition process between **1a** and **2a** is summarized in Table 1. Several bases were examined. When **1a** reacted with **2a** in the presence of Cs_2_CO_3_ as a base in xylene (140 °C, 16 h) in the dark, the reaction proceeded smoothly to generate the desired product **3a** in 72% yield. Changing the base to K_2_CO_3_ decreased the yield to 54%. When Et_3_N, DBU, or no base was used, product **3a** was not obtained (Table 1, entries 1–5). Changing the solvent to DMSO and DMF led to traces of **3a** or no product, respectively (Table 1, entries 6–7). Various molar ratios of **1a** to **2a** were also studied. When the molar ratio of **1a** to **2a** was 1:1, the yield (48%) was much lower than that obtained with a ratio of 1:2 (72%) (Table 1, entries 1 and 8). Changing the quantity of Cs_2_CO_3_ to 2.0 equiv decreased the yield to 56%, but increasing the amount of Cs_2_CO_3_ to 4.5 equiv led to only a slightly higher yield (73%) (Table 1, entries 9 and 10).

**Table 1:** Screening for optimal reaction conditions.^a^

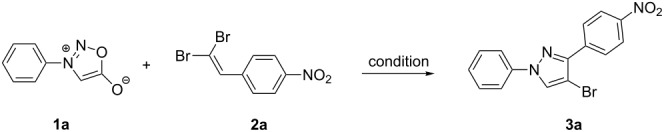				

entry	base (3 equiv)	solvent	T (°C)	yield of 3a (%)^b^

1	Cs_2_CO_3_	xylene	140	72
2	K_2_CO_3_	xylene	140	54
3	Et_3_N	xylene	140	0
4	DBU	xylene	140	0
5	none	xylene	140	0
6	Cs_2_CO_3_	DMSO	140	trace
7	Cs_2_CO_3_	DMF	140	0
8^c^	Cs_2_CO_3_	xylene	140	48
9^d^	Cs_2_CO_3_	xylene	140	56
10^e^	Cs_2_CO_3_	xylene	140	73
**11**	**Cs_2_CO_3_**	**xylene**	**160**	**80**
12	Cs_2_CO_3_	xylene	120	63
13	Cs_2_CO_3_	xylene	90	31

^a^Reaction conditions: 1.0 equiv of **1a** and 2.0 equiv of **2a** were stirred in the dark for 16 h. ^b^Isolated yield. ^c^1.0 equiv of **1a** and 1.0 equiv of **2a.** ^d^2.0 equiv of Cs_2_CO_3_. ^e^4.5 equiv of Cs_2_CO_3_.

The effects of the reaction temperature on the formation of **3a** were also remarkable. At 120 °C and 90 °C, the yields were decreased to 63% and 31%, respectively (Table 1, entries 12–13). At 160 °C, product **3a** was formed in 80% yield (Table 1, entry 11). Ultimately, the optimal reaction conditions were determined as 1:2 molar ratio of **1a** to **2a**, 3.0 equiv Cs_2_CO_3_ base, xylene solvent, 160 °C, and 16 h in the dark (Table 1, entry 11).

Under the optimized conditions, a series of 3-arylsydnones **1** and 2-aryl-1,1-dihalo-1-alkenes **2** substrates were examined. Table 2 shows that in most cases, the desired pyrazoles **3** were smoothly generated in high yields (Table 2, entries 1, 4, 5, and 7–9). In cases with the aromatic portion of 3-arylsydnones **1** carrying either an electron-withdrawing group, such as in chlorine **1d**, or an electron-donating substituent, as in methyl **1b** and methoxyl **1c**, the reactions all proceeded smoothly in moderate to excellent yields. Higher yields were obtained when the aromatic portion of 3-arylsydnones **1** carried an electron-donating group. The presence of a strong electron-donating group in the aromatic portion of 3-arylsydnones **1c** greatly increased the reaction yield (Table 2, entries 7–9). On the other hand, the presence of an electron-withdrawing substituent on the aromatic portion of 3-arylsydnones **1d** lead to the reactions providing pyrazoles **3** in relatively low yields (Table 2, entries 10–12). The effects of 2-aryl-1,1-dihalo-1-alkenes **2** on the formation of pyrazoles **3** were also remarkable. In the cases of the aromatic portion of 2-aryl-1,1-dihalo-1-alkenes **2** carrying nitryl **2a** or cyano **2c** groups all reactions proceeded smoothly in moderate to excellent yields. However, the stronger electron-withdrawing nitryl group provided pyrazoles **3** in relatively high yields. Under the same conditions, the reactivity of 1-(2,2-dibromovinyl)-4-nitrobenzene **2a** was higher than that of 1-(2,2-dichlorovinyl)-4-nitrobenzene **2b** (Table 2, entries 1, 2, 4, 5, 7, 8, 10 and 11). Generally speaking, where present the electron-donating groups of the substrates **1** exhibited stronger electron-donating effects, relative to substrates **2**, leading to higher yields. We also used 1-(2,2-dibromovinyl)-4-methylbenzene (**2d**) as a dipolarophile to react with 3-phenylsydnone. In the process, two isomers, 4-bromo-1-phenyl-3-*p*-tolyl-1*H*-pyrazole (**3m**) and 3-bromo-1-phenyl-4-*p*-tolyl-1*H*-pyrazole (**3n**) were found, and they were generated in 35% and 17% yields, respectively. The experimental results indicate that the electron-donating group, i.e., methyl on the aromatic portion of 2-aryl-1,1-dihalo-1-alkenes, reduced the yields and regioselectivity of the reaction. Products **3** (except **3m** and **3n**) were generated with excellent regiocontrol. It may be that the large substituent (aryl) on the alkyne and the strong electron-withdrawing group, i.e., nitryl or cyano, are of great benefit to the regioselectivity of the reaction.

**Table 2:** Synthesis of 1,3-diaryl-4-halo-1*H*-pyrazoles **3**.^a^

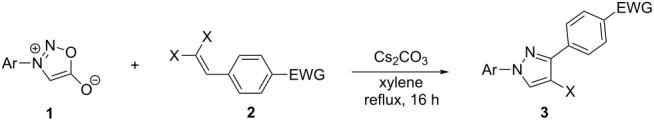				

entry	sydnones **1**	1,1-dihaloalkenes **2**	pyrazoles **3**	yield of **3** (%)^b^

**1**	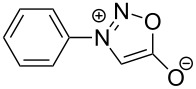 **1a**	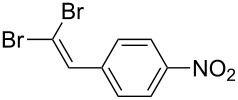 **2a**	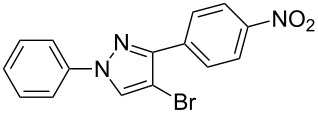 **3a**	80
**2**	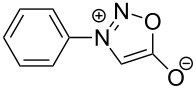 **1a**	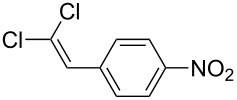 **2b**	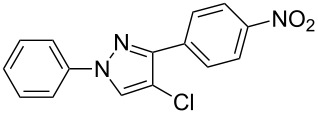 **3b**	65
**3**	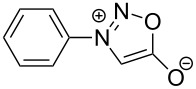 **1a**	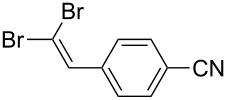 **2c**	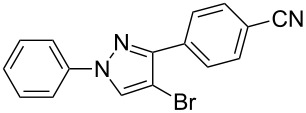 **3c**	56
**4**	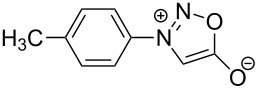 **1b**	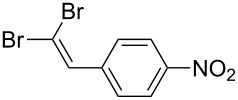 **2a**	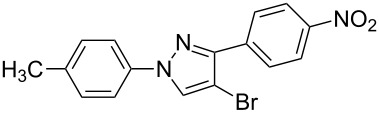 **3d**	84
**5**	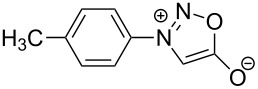 **1b**	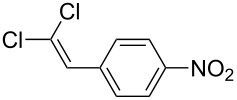 **2b**	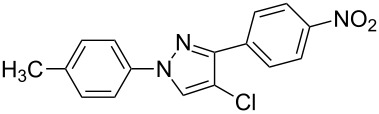 **3e**	73
**6**	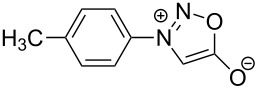 **1b**	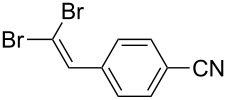 **2c**	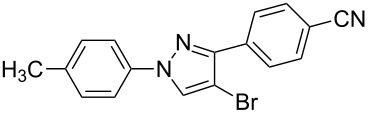 **3f**	63
**7**	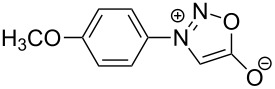 **1c**	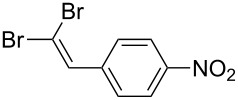 **2a**	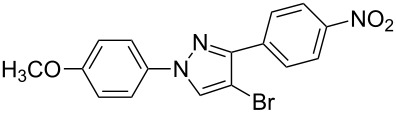 **3g**	92
**8**	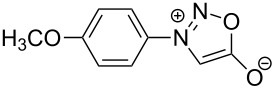 **1c**	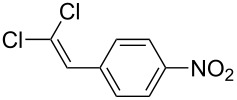 **2b**	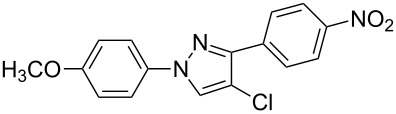 **3h**	75
**9**	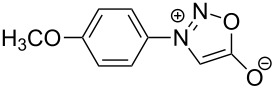 **1c**	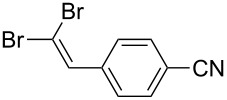 **2c**	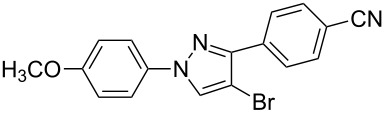 **3i**	72
**10**	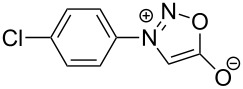 **1d**	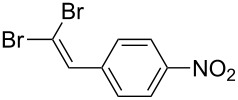 **2a**	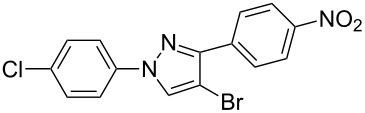 **3j**	66
**11**	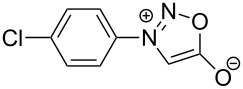 **1d**	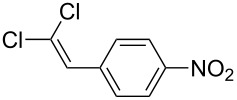 **2b**	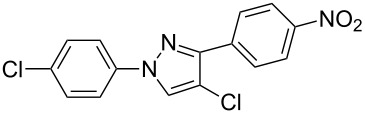 **3k**	49
**12**	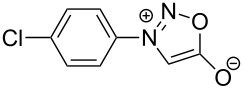 **1d**	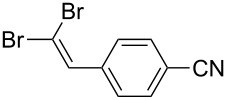 **2c**	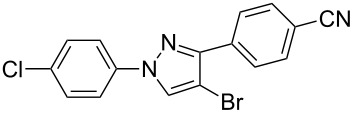 **3l**	41

^a^Reaction conditions: A mixture of **1** (0.3 mmol), **2** (0.6 mmol), and Cs_2_CO_3_ (0.9 mmol) was stirred in 3 mL of xylene in a sealed tube at 160 °C for 16 h in the dark. ^b^Isolated yield.

**Figure 1:**
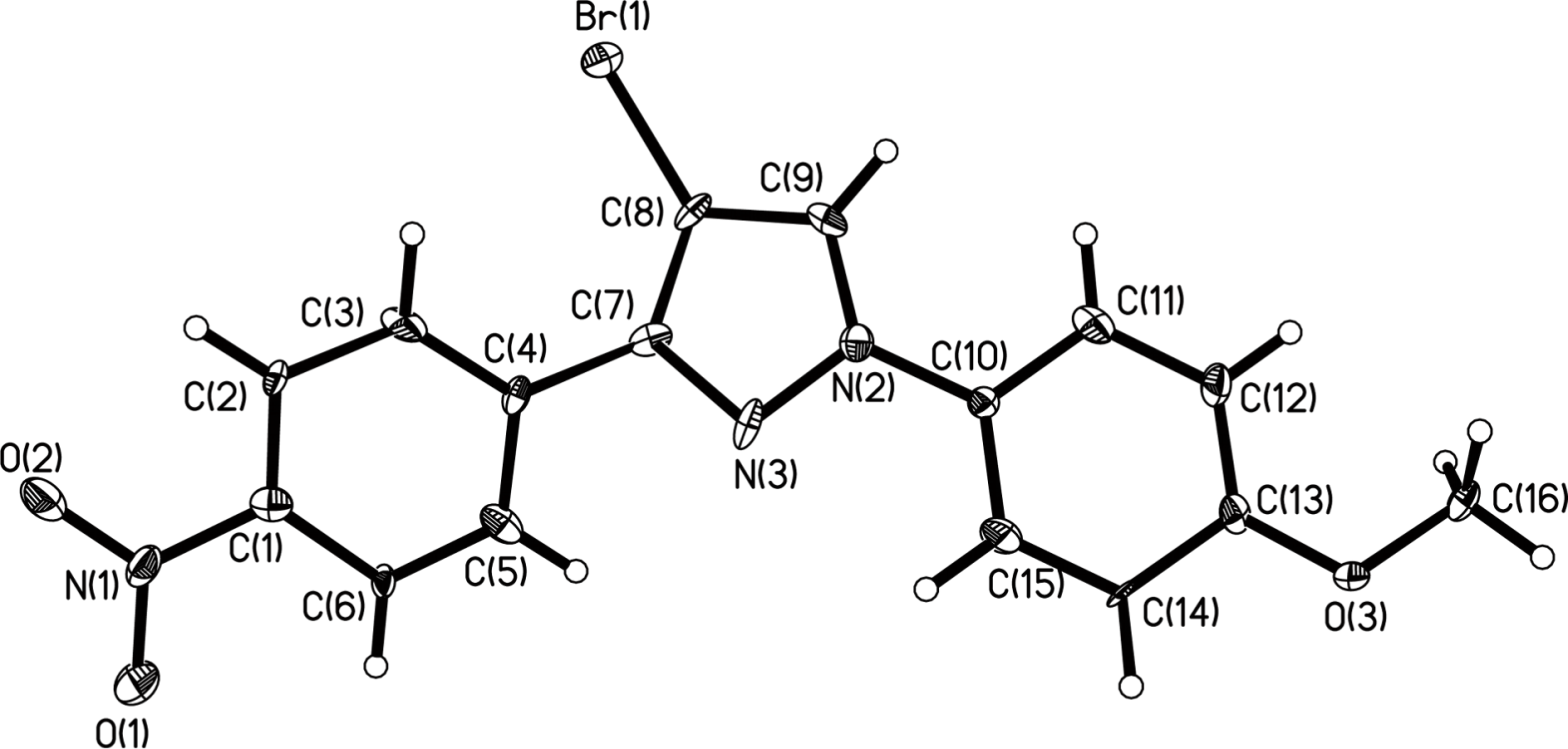
Crystal structure of pyrazole **3g**.

The polysubstituted pyrazoles **3** were characterized by NMR, IR and HRMS. The structure of pyrazole **3g** was further confirmed by single-crystal X-ray diffraction studies (Figure 1).

**Scheme 2:**
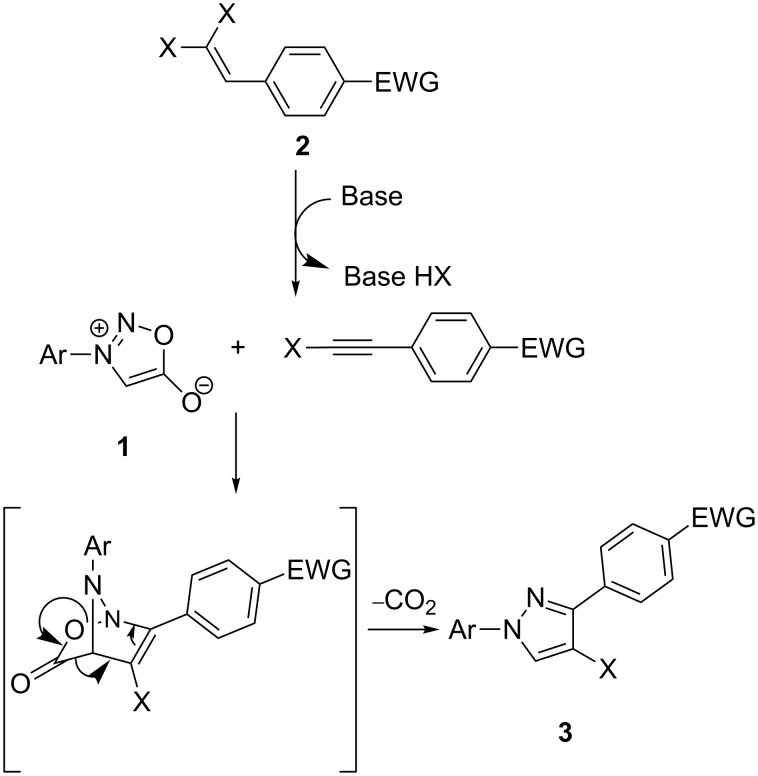
Proposed mechanism for the synthesis of **3**.

In order to elucidate the probable reaction mechanism, a mixture of **1a**, **2a** and xylene was stirred in the dark at 160 °C. After 16 h, no product was found. The results indicate that it was difficult for 2-aryl-1,1-dihalo-1-alkenes to react with 3-arylsydnone in the absence of a base. The steric hindrance of the two halogen atoms on the alkenes is possibly the key factor. Thus a plausible mechanism for the synthesis of pyrazoles **3** could be as follows (Scheme 2): Initially, a haloalkyne is obtained by the elimination of a hydrogen halide from 2-aryl-1,1-dihalo-1-alkenes **2** with Cs_2_CO_3_ as a base [36]. Then, haloalkyne reacts with 3-arylsydnone **1** in a 1,3-dipolar cycloaddition reaction. Finally, CO_2_ is lost [18,34] and pyrazole **3** is generated.

**Scheme 3:**
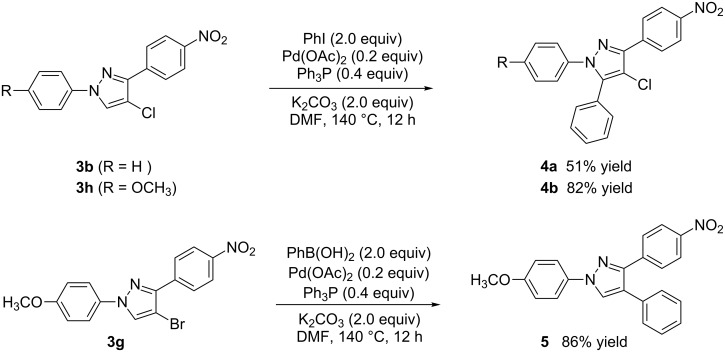
Arylation reactions of pyrazoles (**3**) with iodobenzene or phenylboronic acid.

Subsequently, the arylation reaction of pyrazoles **3** with iodobenzene or phenylboronic acid was investigated (Scheme 3).

Compounds **3b**, **3h,** and **3g** were chosen as the model substrates. When compounds **3b** and **3h** were treated with 2 equiv each of iodobenzene and potassium carbonate in DMF with Pd(OAc)_2_ as a catalyst, the desired 5-aryl substituted products **4a** and **4b** were generated in 51% and 82% yields, respectively. When pyrazole **3g** was treated with 2 equiv each of phenylboronic acid and potassium carbonate in DMF with Pd(OAc)_2_ as a catalyst, the desired 4-aryl substituted product **5** was formed in 86% yield. The three reactions mentioned above indicate that pyrazoles **3** are important intermediates that can easily be converted into 1,2,4-triaryl- or 1,2,5-triaryl-substituted pyrazoles. In addition, 1-(4-methoxyphenyl)-1*H*-pyrazoles (such as compounds **3g**, **3h** and **3i**) are also important intermediates, as they can react with cerium(IV) ammonium nitrate (CAN), leading to N-dearylation followed by the generation of the parent *NH*-pyrazole and *p*-benzoquinone [37].

## Conclusion

In summary, a series of novel 1,3-diaryl-4-halo-1*H*-pyrazoles was synthesized in moderate to excellent yields by using 3-arylsydnones and 2-aryl-1,1-dihalo-1-alkenes in the presence of the mild base Cs_2_CO_3_. The synthesis of a series of new 1,3,4-trisubstituted pyrazoles, which are important heterocycle compounds in medical and pesticide research, was convenient and efficient.

## Supporting Information

File 1 Experimental details and characterization data for all compounds.
