# Supplementary material for: Efficient synthesis of 1,3-diaryl-4-halo-1H-pyrazoles from 3-arylsydnones and 2-aryl-1,1-dihalo-1-alkenes
Source: Beilstein J Org Chem. 2011 Dec 12;7:1656–62. doi: 10.3762/bjoc.7.195 (PMC3252870; doi:10.3762/bjoc.7.195)
Supplement: File 1 — Experimental details and characterization data for all compounds. [file Beilstein_J_Org_Chem-07-1656-s001.pdf]

Supporting Information

for

Efficient synthesis of 1,3-diaryl-4-halo-1*H*-pyrazoles from 3-arylsydnones and 2-aryl-1,1-dihalo-1-alkenes

Yiwen Yang[1,2], Chunxiang Kuang*[1], Hui Jin[1], Qing Yang[3] and Zhongkui Zhang[1]

Address: [1]Department of Chemistry, Tongji University, Siping Road 1239, Shanghai 200092, China; [2]College of Biological, Chemical Sciences and Engineering, Jiaxing University, Jiaxing 314001, China and [3]Department of Biochemistry, School of Life Sciences, Fudan University, Handan Road 220, Shanghai 200433, China

Email: Yiwen Yang - yangyiwen1002@126.com; Chunxiang Kuang* - kuangcx@tongji.edu.cn; Qing Yang - yangqing68@fudan.edu.cn

* Corresponding author

Experimental details and characterization data for all compounds

1. General considerations

All commercially available reagents and solvents were obtained from commercial providers and used without further purification. Melting points were recorded by using a WRS-2A melting point apparatus and were uncorrected. IR spectra were obtained on a Nexus FT-IR spectrophotometer. ^{1}H NMR and ^{13}C NMR spectra were recorded on a Bruker Avance 400 MHz spectrometer. Chemical shifts were reported relative to internal tetramethylsilane (0.00 ppm) for ^{1}H and $CDCl_3$ (77.0 ppm) for ^{13}C. High resolution mass spectra were determined by using a Finnigan-NAT GC/MS/DS 8430 spectrometer. Single crystal X-ray analysis: A representative crystal was surveyed on a Bruker APEX diffractometer. All crystallographic calculations were facilitated by the SHELXL-97 system. Flash column chromatography was performed on 300–400 mesh silica gel. 3-Arylsydnones were prepared according to literature procedures [1,2].

2. General procedure for the preparation of 2-aryl-1,1-dihalo-1-alkenes [3,4]

2.1 Preparation for 2a and 2c

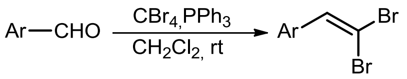

To a solution of triphenylphosphine (20 mmol) and CBr_4 (10 mmol) in CH_2Cl_2 (20 mL) was added ArCHO (5 mmol) in portions at around 0 °C (ice bath). Then the cooling bath was removed and the reaction mixture was stirred at rt until the reaction was complete (monitored by TLC). The reaction was then quenched with petroleum ether and the deposited material was filtered off and washed with ethyl acetate. The solvent was evaporated and the residue was purified by column chromatography (EtOAc/petroleum ether 1:20) to give 1-(2,2-dibromovinyl)-4-nitrobenzene **2a**, 4-(2,2-dibromovinyl)benzonitrile **2c** and 1-(2,2-dibromovinyl)-4-methylbenzene **2d** in 85%, 82% and 90% yields, respectively**.**

2.2 Preparation for 2b

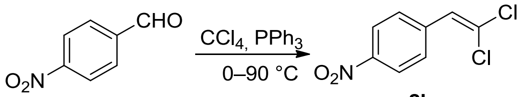

2b

To a solution of triphenylphosphine (39.68 mmol) in CCl_4 (20 mL) was added 4-nitrobenzaldehyde (9.92 mmol) in portions at around 0 °C (ice bath). Then the cooling bath was removed and the reaction mixture was additionally stirred at reflux overnight. After being cooled to rt, the mixture was quenched with petroleum ether and the deposited material was filtered off and washed with ethyl acetate. The solvent was evaporated and the residue was purified by column chromatography (EtOAc/petroleum ether = 1:20) to afford 1-(2,2-dichlorovinyl)-4-nitrobenzene **2b** in 68%yield.

Data of compounds 2

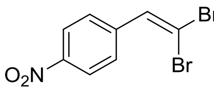

2a

1-(2,2-dibromovinyl)-4-nitrobenzene (2a) [5].

^{1}H NMR (400 MHz, $CDCl_3$): δ = 7.58 (s, 1H), 7.72 (d, *J* = 8.8 Hz, 2H), 8.25 (d, *J* = 8.8 Hz, 2H).

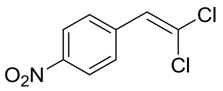

2b

1-(2,2-dichlorovinyl)-4-nitrobenzene (2b) [6].

^{1}H NMR (400 MHz, $CDCl_3$): δ = 6.96 (s, 1H), 7.72 (d, *J* = 8.4 Hz, 2H), 8.25 (d, *J* = 8.8 Hz, 2H).

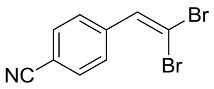

2c

4-(2,2-dibromovinyl)benzonitrile (2c) [5].

^{1}H NMR (400 MHz, $CDCl_3$): δ = 7.52 (s, 1H), 7.65 (d, *J* = 8.8 Hz, 2H), 7.68 (d, *J* = 8.8 Hz, 2H).

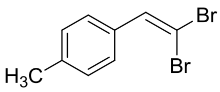

2d

1-(2,2-dibromovinyl)-4-methylbenzene (2d) [5].

^{1}H NMR (400 MHz, $CDCl_3$): δ = 7.22 (d, *J* = 8.0 Hz, 2H), 7.49 (d, *J* = 7.6 Hz, 2H), 7.49 (s, 1H).

3. General procedure for the synthesis of 3

A mixture of 3-arylsydnones (0.3 mmol), 2-aryl-1,1-dihalo-1-alkenes (0.6 mmol), and Cs_2CO_3 (0.9 mmol) in 3 mL xylene was placed in a sealed tube. The tube was heated at 160 °C for 16 h in the dark by using an oil bath. After the reaction was complete (as monitored by TLC), the mixture was cooled to rt. Then the solvent was evaporated in vacuo. The resulting residue was purified by flash column chromatography (petroleum ether/ethyl acetate 50:1, v/v) to yield 1,3-diaryl-4-halo-1*H*-pyrazoles **3**.

Data of compounds 3

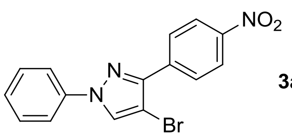

3a

4-Bromo-3-(4-nitrophenyl)-1-phenyl-1*H*-pyrazole (3a), yellow solid, mp:141.3–142.0 °C.
^{1}H NMR (400 MHz, $CDCl_3$): δ = 7.39 (t, *J* = 7.4 Hz, 1H), 7.53 (t, *J* = 8.0 Hz, 2H), 7.75 (d, *J* = 7.6 Hz, 2H), 8.09 (s, 1H), 8.26 (d, *J* = 9.2 Hz, 2H), 8.33 (d, *J* = 9.2 Hz, 2H); ^{13}C NMR (100 MHz, $CDCl_3$): δ = 94.9, 119.1 (2C), 123.7 (2C), 127.6, 128.1 (2C), 129.5, 129.7 (2C), 138.1, 139.3, 147.5, 147.5; IR (KBr): 3152, 3066, 3049, 1598, 1514, 1498, 1464, 1346, 1063, 981, 957, 855, 795, 760, 704, 687, 504 cm^{-1}; HR MS (ESI): *m*/*z* calcd for $C_{15}H_{10}{}^{79}BrN_3O_2$: 343.0012 $[M]^+$; found: 343.0008.

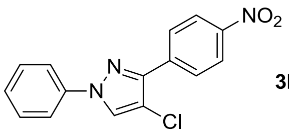

3b

4-chloro-3-(4-nitrophenyl)-1-phenyl-1*H*-pyrazole (3b), yellow solid, mp:140.5−141.1 °C.
^{1}H NMR (400 MHz, $CDCl_3$): δ = 7.39 (t, *J* = 7.4 Hz, 1H), 7.53 (t, *J* = 8.0 Hz, 2H), 7.75 (d, *J* = 7.6 Hz, 2H), 8.07 (s, 1H), 8.27 (d, *J* = 8.8 Hz, 2H), 8.34 (d, *J* = 9.2 Hz, 2H); ^{13}C NMR (100 MHz, $CDCl_3$): δ =111.1, 119.0 (2C), 123.8 (2C), 127.2, 127.6, 127.8 (2C), 129.7 (2C), 137.8, 139.3, 145.9, 147.5; IR (KBr): 3158, 3086, 3050, 1598, 1527, 1514, 1501, 1465, 1457, 1384, 1338, 1064, 994, 957, 855, 791, 757, 703, 685 cm^{-1}; HR MS (ESI): *m*/*z* calcd for $C_{15}H_{10}{}^{35}ClN_3O_2$: 299.0468 $[M]^+$; found: 299.0470.

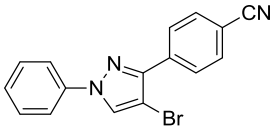

3c

4-(4-bromo-1-phenyl-1*H*-pyrazol-3-yl)benzonitrile (3c), light-yellow solid, mp:131.3–132.8 °C.
^{1}H NMR (400 MHz, $CDCl_3$): δ = 7.36 (t, *J* = 7.4 Hz, 1H), 7.49 (t, *J* = 8.0 Hz, 2H), 7.71 (d, *J* =7.6 Hz, 2H), 7.73 (d, *J* =8.4 Hz, 2H), 8.05 (s, 1H), 8.16 (d, *J* = 8.4 Hz, 2H); ^{13}C NMR (100 MHz, $CDCl_3$): δ = 94.7, 111.8, 119.0 (2C), 125.4, 127.5, 128.0 (2C), 129.4, 129.7 (2C), 132.2 (2C), 136.3, 139.3, 147.8; IR (KBr): 3139, 3076, 3050, 2222, 1599, 1513, 1498, 1464, 1441, 1384, 1336, 1273, 1066, 976, 955, 854, 756, 683, 552 cm^{-1}; HR MS (ESI): *m*/*z* calcd for $C_{16}H_{10}{}^{79}BrN_3$: 323.0072 $[M]^+$; found: 323.0068.

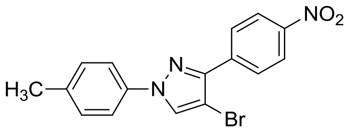

3d

4-bromo-3-(4-nitrophenyl)-1-*p*-tolyl-1*H*-pyrazole (3d), yellow solid, mp:135.0–135.7 °C.
^{1}H NMR (400 MHz, $CDCl_3$): δ = 2.41 (s, 3H), 7.29 (d, *J* = 8.4 Hz, 2H), 7.60 (d, *J* = 8.4 Hz, 2H), 8.02(s, 1H), 8.24 (d, *J* = 8.8 Hz, 2H), 8.31 (d, *J* = 8.8 Hz, 2H); ^{13}C NMR (100 MHz, $CDCl_3$): δ = 21.0, 94.6, 119.1 (2C), 123.7 (2C), 128.1 (2C), 129.5, 130.2 (2C), 137.1, 137.6, 138.2, 147.2, 147.4; IR (KBr): 3130, 2953, 2924, 2854, 1598, 1525, 1500, 1460, 1377, 1336, 1071, 975, 957, 855, 811, 756, 698, 506 cm^{-1}; HR MS (ESI): *m*/*z* calcd for $C_{16}H_{12}{}^{79}BrN_3O_2$: 357.0072 $[M]^+$; found: 357.0068.

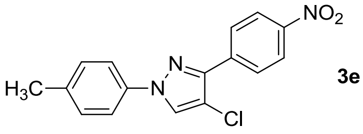

3e

4-chloro-3-(4-nitrophenyl)-1-*p*-tolyl-1*H*-pyrazole (3e), yellow solid, mp:168.2–169.2 °C.
^{1}H NMR (400 MHz, $CDCl_3$): δ = 2.44 (s, 3H), 7.32 (d, *J* = 8.4 Hz, 2H), 7.62 (d, *J* = 8.8 Hz, 2H), 8.02 (s, 1H), 8.27 (d, *J* = 8.8 Hz, 2H), 8.33 (d, *J* = 8.8 Hz, 2H); ^{13}C NMR (100 MHz, $CDCl_3$): δ = 21.0, 110.8, 119.0 (2C), 123.8 (2C), 127.1, 127.8 (2C), 130.2 (2C), 130.8, 137.6, 137.9, 145.7, 147.4; IR (KBr): 3148, 2953, 2924, 2854, 1600, 1529, 1508, 1457, 1383, 1337, 1065, 991, 957, 858, 814, 782, 719, 698 cm^{-1}; HR MS (ESI): *m*/*z* calcd for $C_{16}H_{12}{}^{35}ClN_3O_2$: 313.0583 $[M]^+$; found: 313.0585.

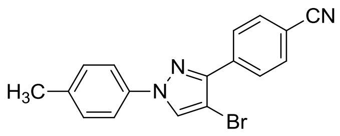

3f

4-(4-bromo-1-*p*-tolyl-1*H*-pyrazol-3-yl)benzonitrile (3f), light-yellow solid, mp:146.8–147.5 °C. ^{1}H NMR (400 MHz, $CDCl_3$): δ = 2.43 (s, 3H), 7.30 (d, *J* = 8.4 Hz, 2H), 7.61 (d, *J* = 8.4 Hz, 2H), 7.75 (d, *J* = 8.4 Hz, 2H), 8.03 (s, 1H), 8.19 (d, *J* = 8.4 Hz, 2H); ^{13}C NMR (100 MHz, $CDCl_3$): δ =21.0, 94.4, 111.7, 119.0 (2C), 125.3, 127.9 (2C), 129.4, 130.2 (2C), 132.2 (2C), 136.4, 137.1, 137.5, 147.5; IR (KBr): 3126, 3041, 2953, 2923, 2854, 2224, 1608, 1521, 1457, 1384, 1338, 1068, 975, 956, 843, 819, 811, 732, 684, 550, 507 cm^{-1}; HR MS (ESI): *m*/*z* calcd for $C_{17}H_{12}{}^{79}BrN_3$: 337.0158 $[M]^+$; found: 337.0160.

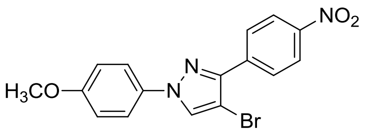

3g

4-bromo-1-(4-methoxyphenyl)-3-(4-nitrophenyl)-1*H*-pyrazole (3g), yellow solid, mp: 178.9–179.3 °C. ^{1}H NMR (400 MHz, $CDCl_3$): δ = 3.86 (s, 3H), 7.00 (d, *J* = 9.2 Hz, 2H), 7.62 (d, *J* = 8.8 Hz, 2H), 7.97 (s, 1H), 8.23 (d, *J* = 8.8 Hz, 2H), 8.30 (d, *J* = 9.2 Hz, 2H); ^{13}C NMR (100 MHz, $CDCl_3$): δ = 55.6, 94.4, 114.7 (2C), 120.8 (2C), 123.7 (2C), 128.0 (2C), 129.6, 133.0, 138.3, 147.0, 147.4, 159.1; IR (KBr): 3147, 3093, 3080, 2954, 2924, 2853, 1599, 1520, 1499, 1459, 1436, 1384, 1336, 1250, 1188, 1108, 1069, 1034, 980, 958, 854, 827, 793, 781, 758, 699, 646, 624, 517 cm^{-1} ; HR MS (ESI): *m*/*z* calcd for $C_{16}H_{12}{}^{79}BrN_3O_3$: 373.0066 $[M]^+$; found: 373.0068.

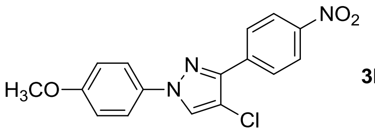

3h

4-chloro-1-(4-methoxyphenyl)-3-(4-nitrophenyl)-1*H*-pyrazole (3h), yellow solid, mp:171.9-172.6 °C. ^{1}H NMR (400 MHz, $CDCl_3$): δ = 3.86 (s, 3H), 7.00 (d, *J* = 9.2 Hz, 2H), 7.61 (d, *J* = 8.8 Hz, 2H), 7.94 (s, 1H), 8.23 (d, *J* = 9.2 Hz, 2H), 8.30 (d, *J* = 9.2 Hz, 2H); ^{13}C NMR (100 MHz, $CDCl_3$): δ =55.6, 110.6, 114.7 (2C), 120.7 (2C), 123.8 (2C), 127.2, 127.7 (2C), 133.0, 137.9, 145.5, 147.4, 159.1; IR (KBr): 3154, 3095, 2954, 2924, 2854, 1600, 1525, 1504, 1462, 1384, 1340, 1250, 1190, 1108, 1069, 1033, 992, 959, 855, 826, 793, 778, 758, 699, 653, 625 cm^{-1}; HR MS (ESI): *m*/*z* calcd for $C_{16}H_{12}{}^{35}ClN_3O_3$: 329.0611 $[M]^+$; found: 329.0612.

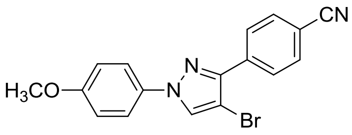

3i

4-(4-bromo-1-(4-methoxyphenyl)-1*H*-pyrazol-3-yl)benzonitrile (3i), yellow solid, mp: 135.7–139.8 °C. ^{1}H NMR (400 MHz, $CDCl_3$): δ = 3.86 (s, 3H), 6.99 (d, *J* = 9.2 Hz, 2H), 7.60 (d, *J* = 8.8 Hz, 2H), 7.73 (d, *J* = 8.4 Hz, 2H), 7.95 (s, 1H), 8.15 (d, *J* = 8.4 Hz, 2H); ^{13}C NMR (100 MHz, $CDCl_3$): δ = 55.6, 94.1, 111.7, 114.7 (2C), 120.8 (2C), 126.9, 127.9 (2C), 129.5, 132.2 (2C), 133.0, 136.4, 147.3, 159.0; IR (KBr): 3139, 2954, 2924, 2854, 2225, 1611, 1518, 1499, 1459, 1377, 1338, 1302, 1250, 1185, 1067, 1034, 978, 959, 842, 828, 796, 733, 690, 652, 573, 550, 518 cm^{-1}; HR MS (ESI): *m*/*z* calcd for $C_{17}H_{12}{}^{79}BrN_3O$: 353.0178 $[M]^+$; found: 353.0181.

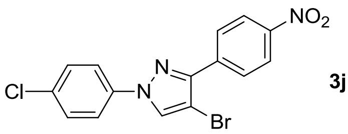

3j

4-bromo-1-(4-chlorophenyl)-3-(4-nitrophenyl)-1*H*-pyrazole (3j), light-yellow solid, mp: 155.9−156.8 °C. ^{1}H NMR (400 MHz, $CDCl_3$): δ = 7.39 (d, *J* = 8.8 Hz, 2H), 7.60 (d, *J* = 8.8 Hz, 2H), 7.97 (s, 1H), 8.15 (d, *J* = 9.2 Hz, 2H), 8.24 (d, *J* = 8.8 Hz, 2H); ^{13}C NMR (100 MHz, $CDCl_3$): δ =95.3, 120.2 (2C), 123.7 (2C), 128.2 (2C), 129.4, 129.8 (2C), 133.2, 133.4, 137.8, 147.6, 147.8; IR (KBr): 3143, 3085, 1598, 1515, 1494, 1426, 1385, 1337, 1062, 1013, 981, 954, 904, 855, 822, 794, 729, 701, 585, 551, 503 cm^{-1}; HR MS (ESI): *m*/*z* calcd for $C_{15}H_9{}^{79}Br^{35}Cl\ N_3O_2$: 376.9582 $[M]^+$; found: 376.9580.

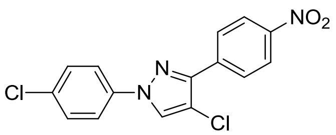

3k

4-chloro-1-(4-chlorophenyl)-3-(4-nitrophenyl)-1*H*-pyrazole (3k), yellow solid, mp: 159.3–160.5 °C. ^{1}H NMR (400 MHz, $CDCl_3$): δ = 7.47 (d, *J* = 8.8 Hz, 2H), 7.67 (d, *J* = 8.8 Hz, 2H), 8.02 (s, 1H), 8.23 (d, *J* = 8.8 Hz, 2H), 8.31 (d, *J* = 8.8 Hz, 2H); ^{13}C NMR (100 MHz, $CDCl_3$): δ =111.5, 120.1 (2C), 123.8 (2C), 127.0, 127.8 (2C), 129.8 (2C), 133.2, 137.5, 137.8, 146.3, 147.6; IR (KBr): 3142, 3073, 1600, 1515, 1498, 1427, 1392, 1337, 1059, 1013, 996, 955, 855, 822, 757, 702, 602 cm^{-1}; HR MS (ESI): *m*/*z* calcd for $C_{15}H_9{}^{35}Cl_2N_3O_2$: 333.0108 $[M]^+$; found: 333.0110.

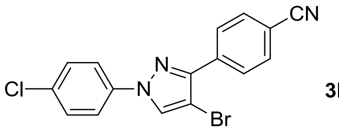

3l

4-(4-bromo-1-(4-chlorophenyl)-1*H*-pyrazol-3-yl)benzonitrile (3l), light-yellow solid, mp:160.2–161.1 °C. ^{1}H NMR (400 MHz, $CDCl_3$): δ = 7.38 (d, *J* = 8.8 Hz, 2H), 7.58 (d, *J* = 8.8 Hz, 2H), 7.66 (d, *J* = 8.8 Hz, 2H), 7.95 (s, 1H), 8.07 (d, *J* = 8.4 Hz, 2H); ^{13}C NMR (100 MHz, $CDCl_3$): δ =95.1, 112.0, 118.8, 120.1 (2C), 128.0 (2C), 129.3, 129.8 (2C), 132.2 (2C), 133.1, 136.0, 137.8, 148.1; IR (KBr): 3135, 3098, 3060, 2227, 1609, 1597, 1514, 1493, 1442, 1424, 1388, 1338, 1309, 1272, 1066, 1016, 980, 955, 839, 822, 732, 688, 597, 548 cm^{-1}; HR MS (ESI): *m*/*z* calcd for $C_{16}H_9{}^{79}Br^{35}ClN_3$: 356.9672 $[M]^+$; found: 356.9668.

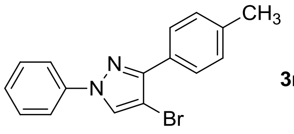

3m

4-bromo-1-phenyl-3-*p*-tolyl-1*H*-pyrazole (3m), tan colored oil. ^{1}H NMR (400 MHz, $CDCl_3$): δ = 2.45 (s, 3H), 7.31 (d, *J* = 7.6 Hz, 2H), 7.35 (d, *J* = 7.2 Hz, 1H), 7.49 (t, *J* = 8.0 Hz, 2H), 7.74 (d, *J* = 8.0 Hz, 2H), 7.93 (d, *J* = 8.0 Hz, 2H), 8.03 (s, 1H); ^{13}C NMR (100 MHz, $CDCl_3$): δ =21.2, 118.9(2C), 124.5, 125.8, 126.0, 127.0, 127.4, 127.8 (2C), 129.4 (2C), 129.5 (2C), 137.4, 139.5; IR (KBr): 3065, 2928, 2855, 1601, 1516, 1498, 1457, 1396, 1220, 1059, 978, 955, 824, 650, 507 cm^{-1}; HR MS (ESI): *m*/*z* calcd for $C_{16}H_{13}{}^{79}BrN_2$: 312.0276 $[M]^+$; found: 312.0285.

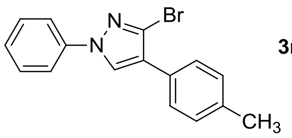

3n

3-bromo-1-phenyl-4-*p*-tolyl-1*H*-pyrazole (3n), tan colored oil.

^{1}H NMR (400 MHz, $CDCl_3$): δ = 2.43 (s, 3H), 7.28 (d, *J* = 8.0 Hz, 2H), 7.34 (d, *J* = 7.4 Hz, 1H), 7.49 (t, *J* = 8.0 Hz, 2H), 7.53 (d, *J* = 8.0 Hz, 2H), 7.72 (d, *J* = 7.6 Hz, 2H), 7.97 (s, 1H); ^{13}C NMR (100 MHz, $CDCl_3$): δ = 21.4, 94.4, 118.9(2C), 126.9, 127.7 (2C), 128.7, 128.9, 129.1 (2C), 129.5 (2C), 138.4, 139.6, 150.1; IR (KBr): 3030, 2930, 2863, 1617, 1576, 1507, 1497, 1465, 1386, 1253, 1062, 989, 929, 827, 648, 517 cm^{-1}; HR MS (ESI): *m*/*z* calcd for $C_{16}H_{13}{}^{79}BrN_2$: 312.0276 $[M]^+$; found: 312.0288.

4. General procedure for the synthesis of 4a and 4b

A mixture of pyrazole **3b** or **3h** (0.2 mmol), $Pd(OAc)_2$ (0.04 mmol), K_2CO_3 (0.4 mmol), PPh_3 (0.08 mmol,) and iodobenzene (0.4 mmol) in 1 mL DMF was placed in a sealed tube. The tube was heated at 140 °C for 12 h in the dark by using an oil bath. After the reaction was completed (as monitored by TLC), the mixture was cooled to rt. Then 30 mL water was added to the reaction mixture, which was extracted with EtOAc (3 × 20 mL). The combined organic layers were washed with saturated brine, dried over anhydrous Na_2SO_4 and concentrated under reduced pressure. The resulting residue was purified by flash column chromatography (petroleum ether/ethyl acetate 20:1, v/v) to yield **4a** or **4b**.

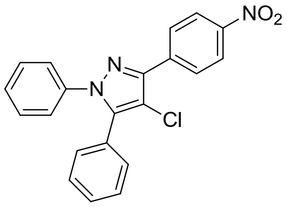

4a

4-chloro-3-(4-nitrophenyl)-1,5-diphenyl-1*H*-pyrazole (4a), yellow solid. mp:135.9–137.0 °C

^{1}H NMR (400 MHz, $CDCl_3$): δ = 7.30–7.40 (m, 7H), 7.40–7.46 (m, 3H), 8.30 (d, *J* = 8.8Hz, 2H), 8.35 (d, *J* = 8.8 Hz, 2H); ^{13}C NMR (100 MHz, $CDCl_3$): δ =109.5, 118.9, 123.8 (2C), 124.8 (2C), 127.8, 128.0 (2C), 128.1, 128.7 (2C), 129.1 (2C), 129.3, 130.0 (2C), 138.1, 139.5, 145.7, 147.5; IR (KBr): 3077, 1599, 1513, 1499, 1457, 1340, 1027, 966, 853, 803, 767, 710, 696 cm^{-1}; HR MS (ESI): *m*/*z* calcd for $C_{21}H_{14}{}^{35}ClN_3O_2$: 375.0772 $[M]^+$; found: 375.0775.

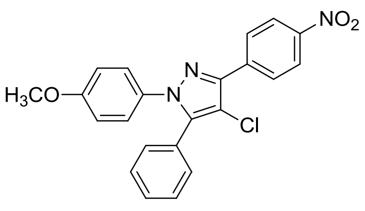

4b

4-chloro-1-(4-methoxyphenyl)-3-(4-nitrophenyl)-5-phenyl-1*H*-pyrazole (4b), reddish-brown solid. mp: 153.2–154.0 °C. ^{1}H NMR(400 MHz, $CDCl_3$): δ = 3.83 (s, 3H), 6.87 (d, *J* = 8.8 Hz, 2H), 7.23 (d, *J* = 8.8 Hz, 2H), 7.32-7.37 (m,2H), 7.40-7.45 (m,3H), 8.29 (d, *J* = 9.2 Hz, 2H), 8.34 (d, *J* = 8.8 Hz, 2H); ^{13}C NMR (100 MHz, $CDCl_3$): δ =55.5, 109.0, 114.2 (2C), 123.8 (2C), 126.3 (2C), 127.8, 128.0 (2C), 128.6 (2C), 129.2, 130.0 (2C), 132.7, 138.2, 141.1, 145.3, 147.4, 159.3; IR (KBr): 3079, 3009, 2954, 2923, 2851, 1600, 1514, 1481, 1465, 1347, 1301, 1251, 1171, 1111, 1077, 1033, 993, 968, 855, 837, 778, 758, 704, 619 cm^{-1}; HR MS (ESI): *m*/*z* calcd for $C_{22}H_{16}{}^{35}ClN_3O_3$: 405.0924 $[M]^+$; found: 405.0926.

5. General procedure for the synthesis of 5

A mixture of pyrazole **3g** (0.2 mmol), $Pd(OAc)_2$ (0.04 mmol), K_2CO_3 (0.4 mmol), PPh_3 (0.08 mmol,) and phenylboronic acid (0.4 mmol) in 1 mL DMF was placed in a sealed tube. The tube was heated at 140 °C for 12 h in the dark by using an oil bath. After the reaction was completed (as monitored by TLC), the mixture was cooled to rt. Then 30 mL water was added to the reaction mixture, which was extracted with EtOAc (3 × 20 mL). The combined organic layers were washed with saturated brine, dried over anhydrous Na_2SO_4 and concentrated under reduced pressure. The resulting residue was purified by flash column chromatography (petroleum ether/ethyl acetate 20:1, v/v) to yield 1-(4-methoxyphenyl)-3-(4-nitrophenyl)-4-phenyl-1*H*-pyrazole (**5**).

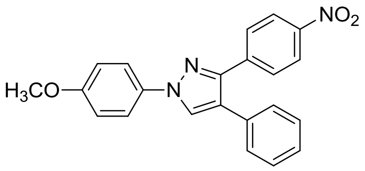

5

1-(4-methoxyphenyl)-3-(4-nitrophenyl)-4-phenyl-1*H*-pyrazole (5), yellow solid. mp: 130.3–131.2 °C. ^{1}H NMR (400 MHz, $CDCl_3$): δ = 3.90 (s, 3H), 7.04 (d, *J* = 8.8 Hz, 2H), 7.32–7.45 (m, 5H), 7.72 (d, *J* = 8.8 Hz, 2H), 7.80 (d, *J* = 8.4 Hz, 2H), 7.96 (s, 1H), 8.19 (d, *J* = 8.8 Hz, 2H); ^{13}C NMR (100 MHz, $CDCl_3$): δ = 55.6, 114.7 (2C), 115.3, 120.9 (2C), 123.6 (2C), 127.5 (2C), 128.7 (2C), 128.8, 128.9 (2C), 129.6, 132.3, 133.4, 139.9, 147.1, 147.4, 158.8; IR (KBr): 3078, 2956, 2922, 2851, 1599, 1551, 1517, 1488, 1463, 1339, 1245, 1215, 1179, 1109, 1063, 1030, 971, 913, 853, 830, 760, 743 cm^{-1}; HR MS (ESI): *m*/*z* calcd for $C_{22}H_{17}N_3O_3$: 371.1275 $[M]^+$; found: 371.1277.

References

1. Rai, N. S.; Kalluraya, B.; Lingappa, B.; Shenoy, S; Puranic, V. G. *Eur. J. Med. Chem.* **2008,** *43,* 1715. doi:10.1016/j.ejmech.2007.08.002
2. Asundaria, S. T.; Patel, N. S.; Patel, K. C. *Org. Commun.* **2010,** *3,* 30.
3. Sun, C.; Xu, B. *J. Org. Chem*. **2008,** *73,* 7361. doi:10.1021/jo801219j
4. Corey, E. J.; Fuchs, P. L. *Tetrahedron Lett.* **1972,** *13,* 3769. doi:10.1016/S0040-4039(01)94157-7
5. Huh, D. H.; Jeong, J. S.; Lee, H. B.; Ryu, H.; Kim, Y. G. *Tetrahedron* **2002,** *58,* 9925. doi:10.1016/S0040-4020(02)01324-8
6. Newman, S. G.; Bryan, C. S.; Perez, D.; Lautens, M. *Synthesis* **2011,** 342. doi:10.1055/s-0030-1258368

NMR spectra data for compounds 2

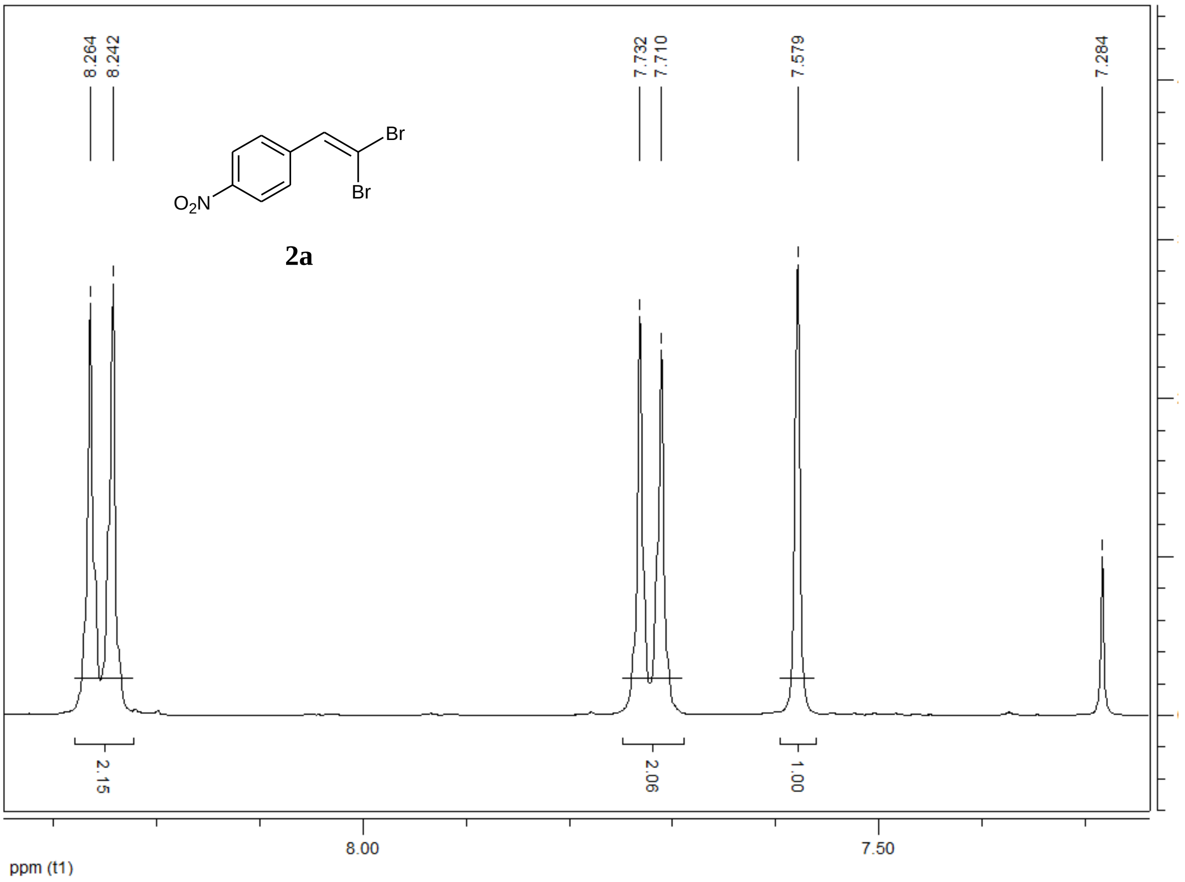

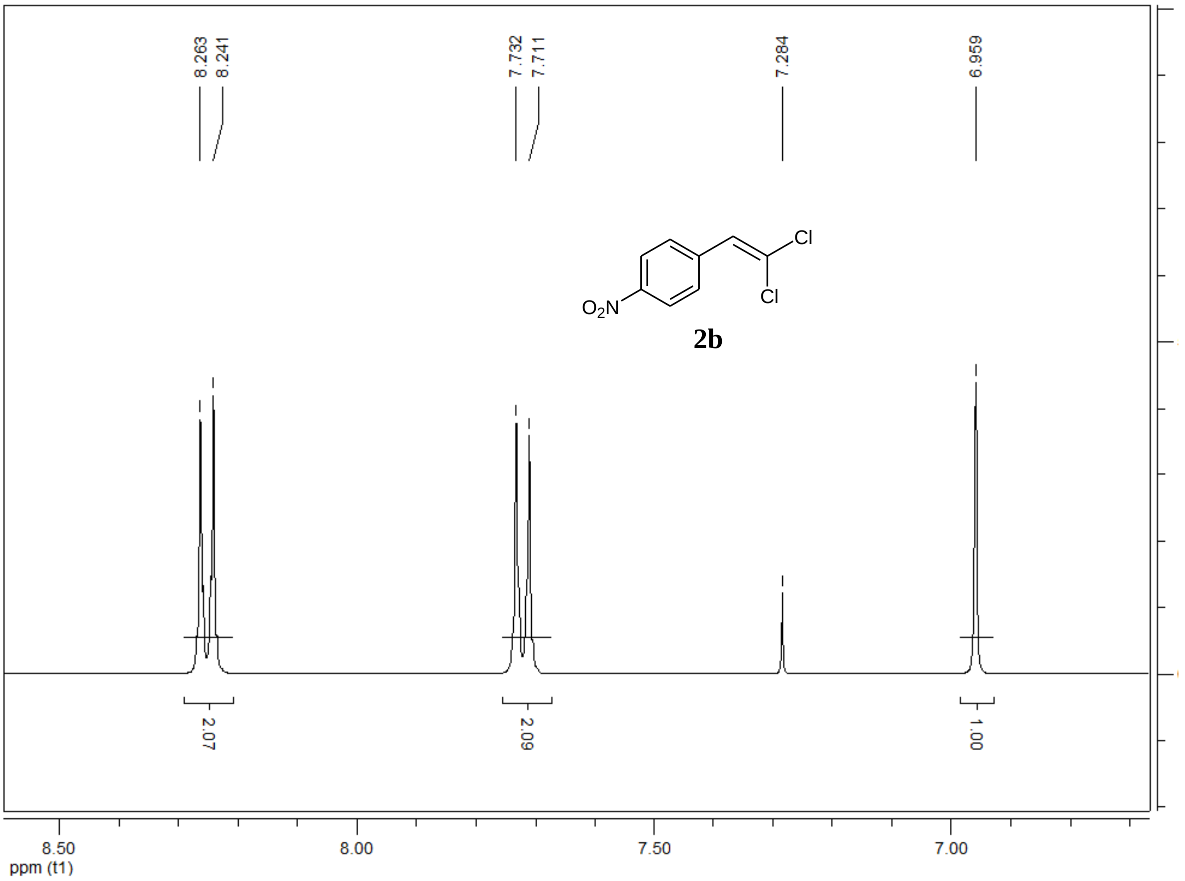

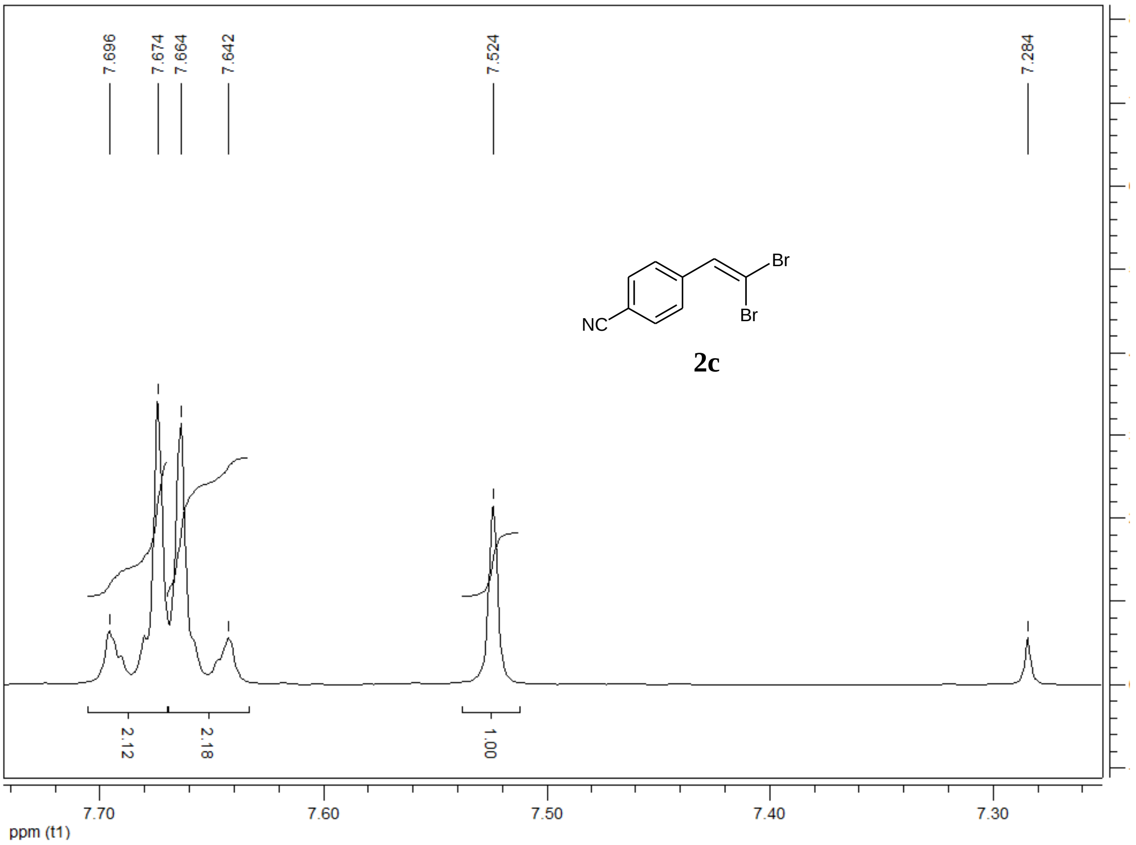
7.696
7.674
7.664
7.642
7.524
7.284
NC
Br
Br
2.12
2.18
1.00
7.70
7.60
7.50
7.40
7.30
ppm (t1)

2c

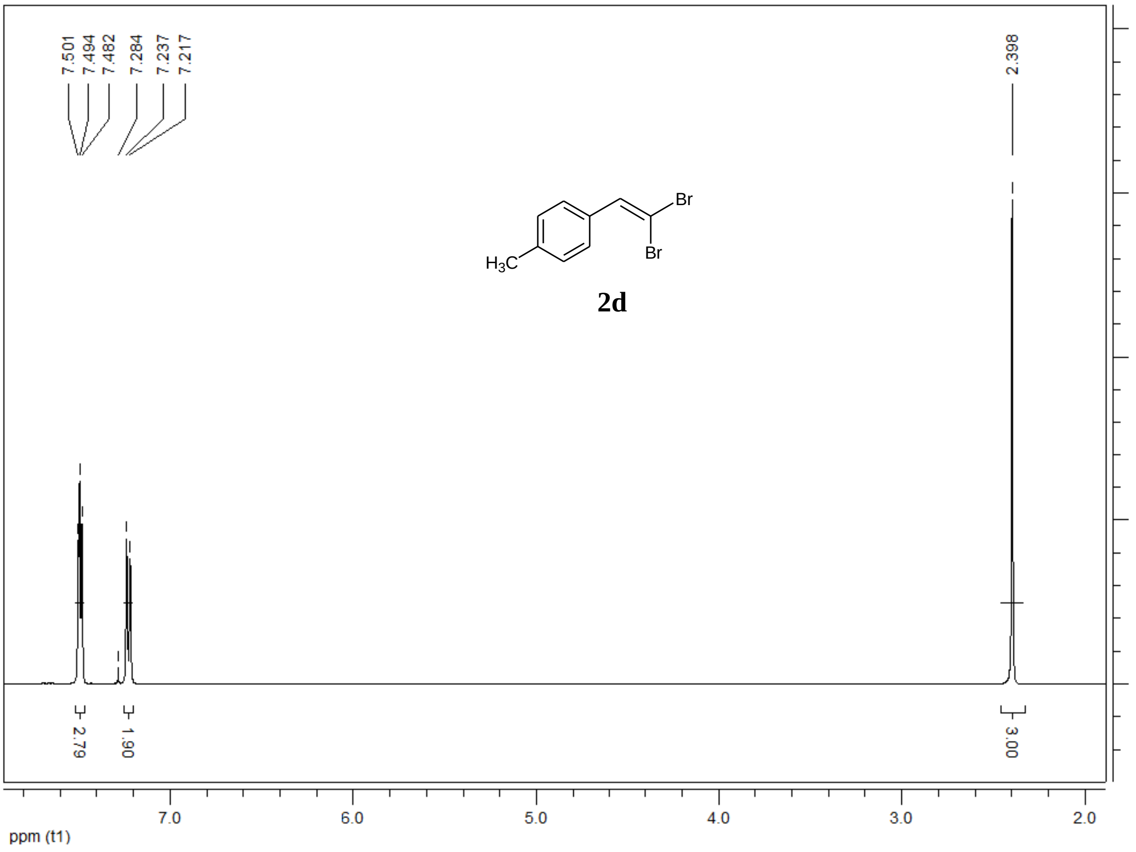
7.501
7.494
7.482
7.284
7.237
7.217
2.398
H_3C
Br
Br
2.79
1.90
3.00
7.0
6.0
5.0
4.0
3.0
2.0
ppm (t1)

2d

NMR spectra data for compounds 3

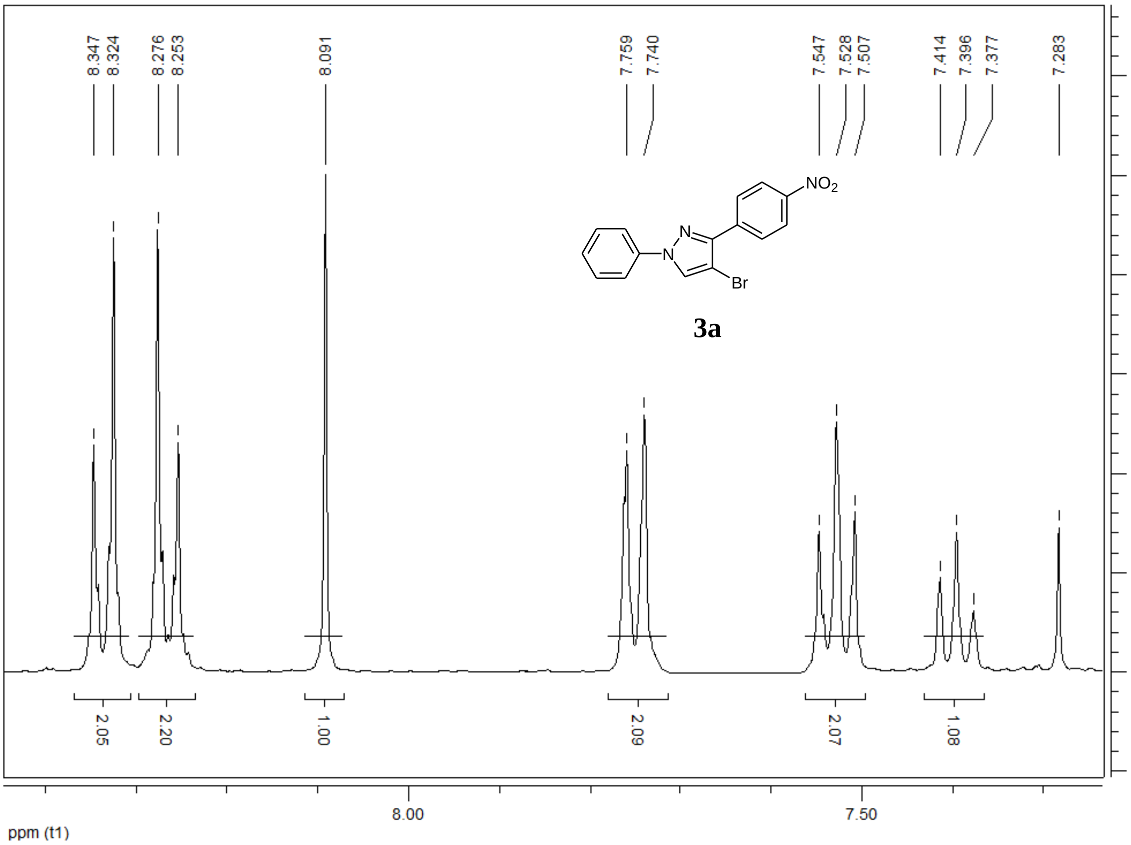

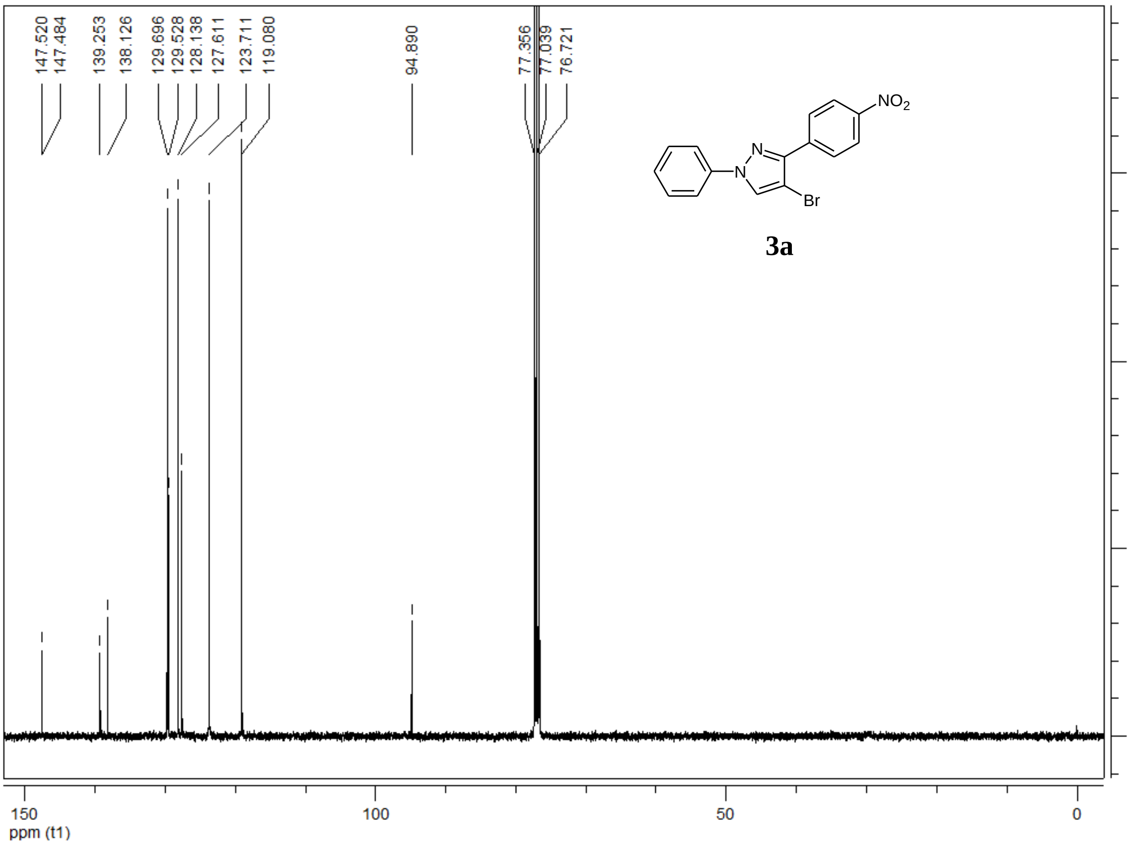

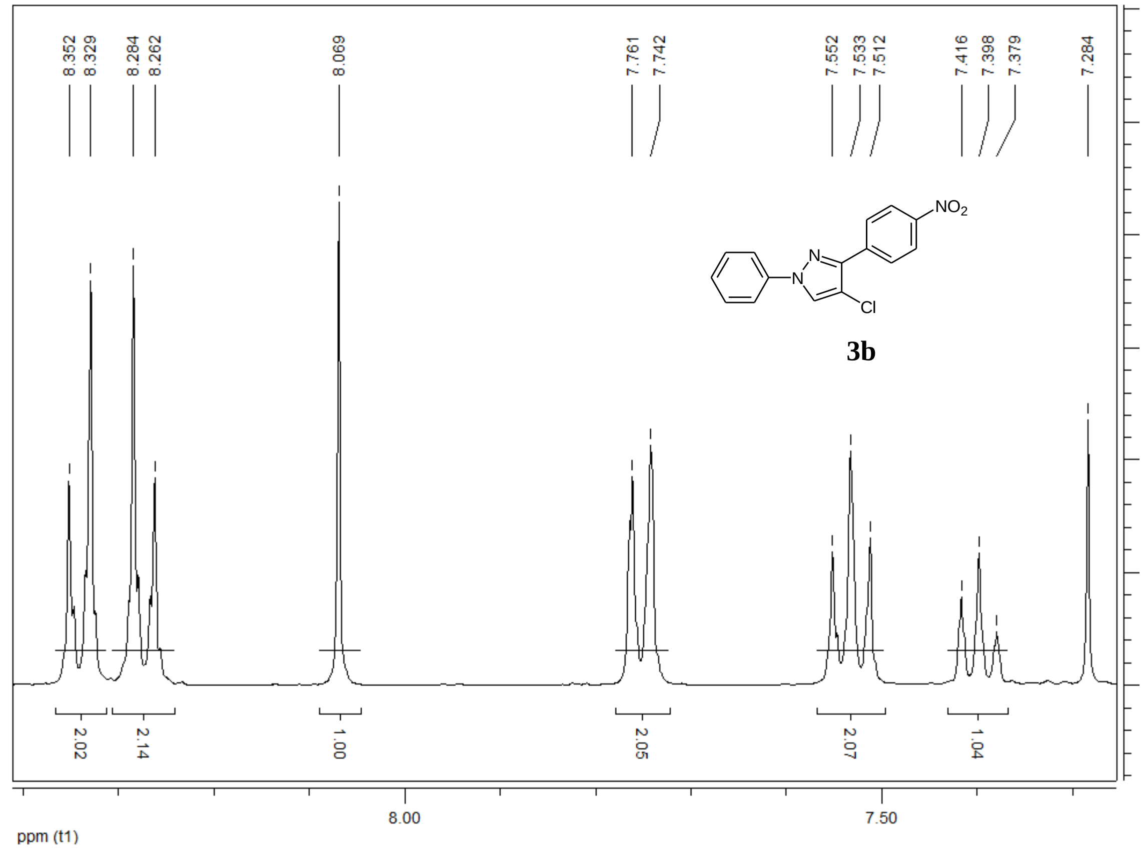
8.352
8.329
8.284
8.262
8.069
7.761
7.742
7.552
7.533
7.512
7.416
7.398
7.379
7.284
NO2
N
N
Cl
3b
2.02
2.14
1.00
2.05
2.07
1.04
8.00
7.50
ppm (t1)

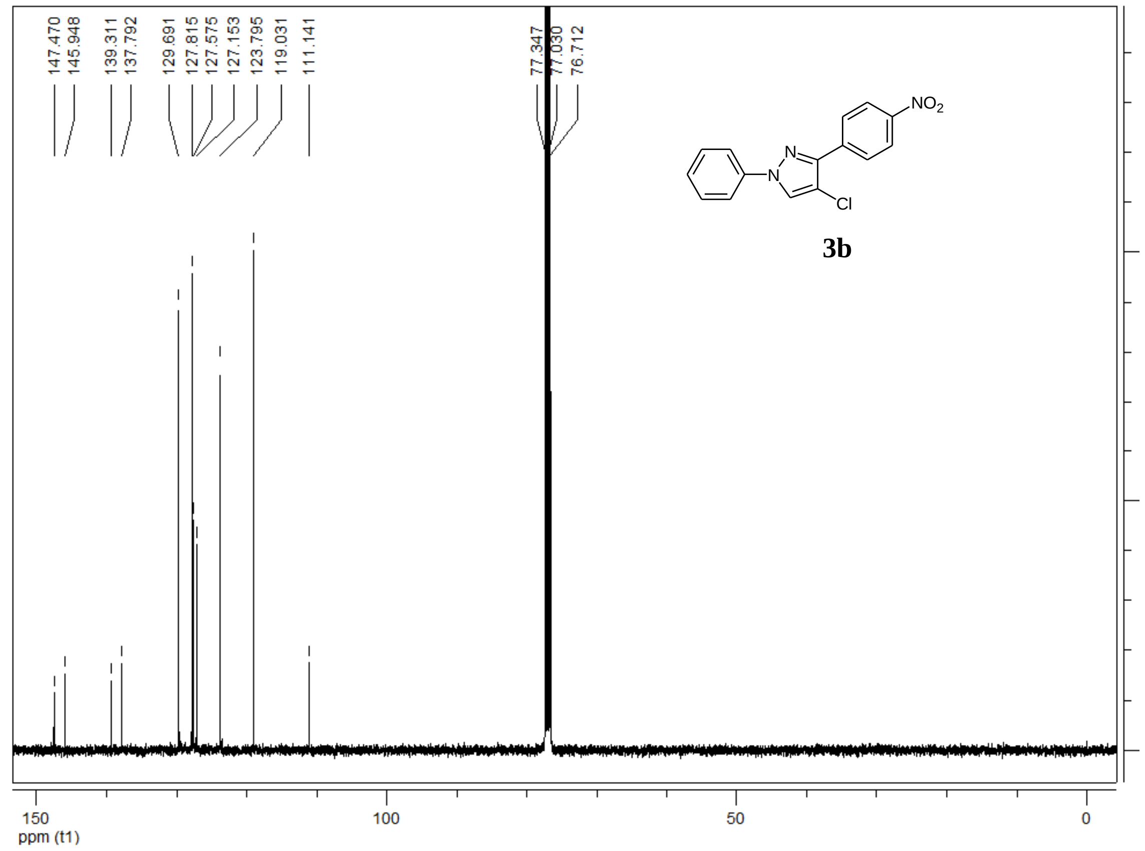
147.470
145.948
139.311
137.792
129.691
127.815
127.575
127.153
123.795
119.031
111.141
77.347
77.030
76.712
NO2
N
N
Cl
3b
150
100
50
0
ppm (t1)

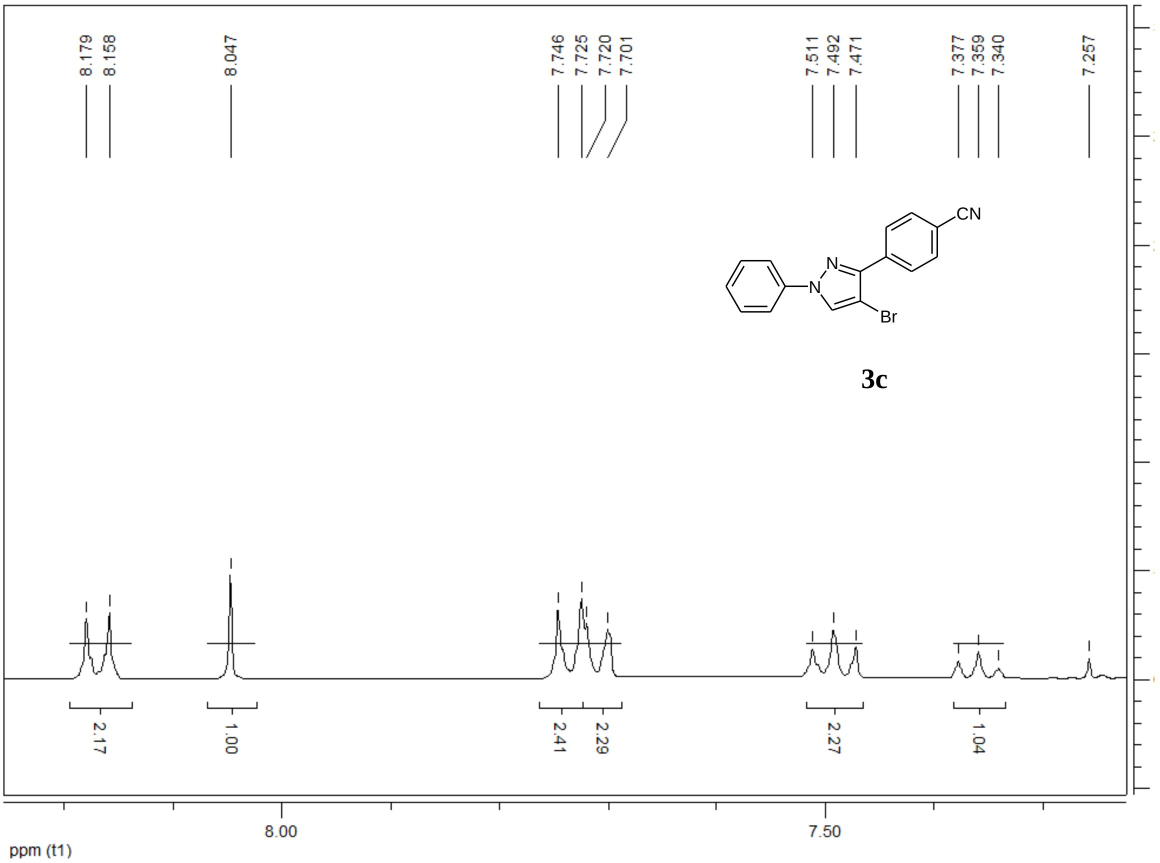
8.179
8.158
8.047
7.746
7.725
7.720
7.701
7.511
7.492
7.471
7.377
7.359
7.340
7.257
CN
N
N
Br
3c
2.17
1.00
2.41
2.29
2.27
1.04
8.00
7.50
ppm (t1)

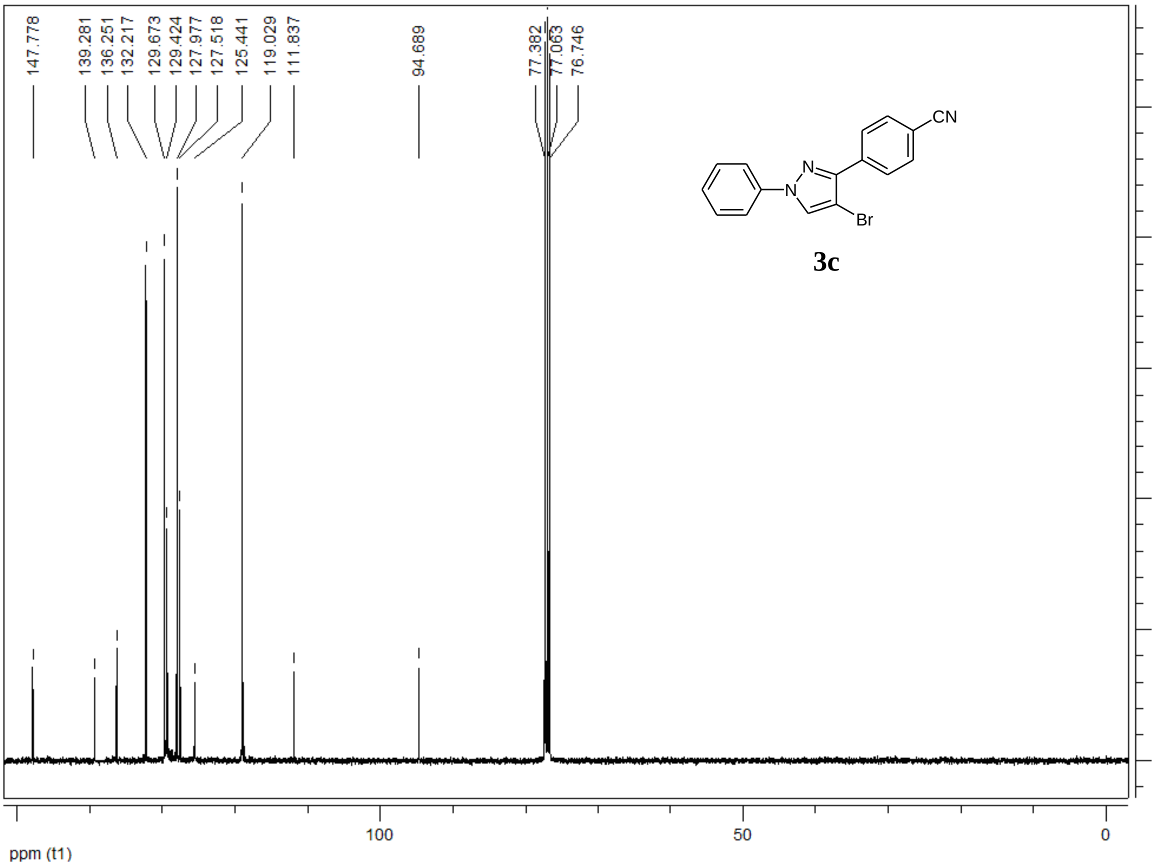
147.778
139.281
136.251
132.217
129.673
129.424
127.977
127.518
125.441
119.029
111.837
94.689
77.382
77.063
76.746
CN
N
N
Br
3c
100
50
0
ppm (t1)

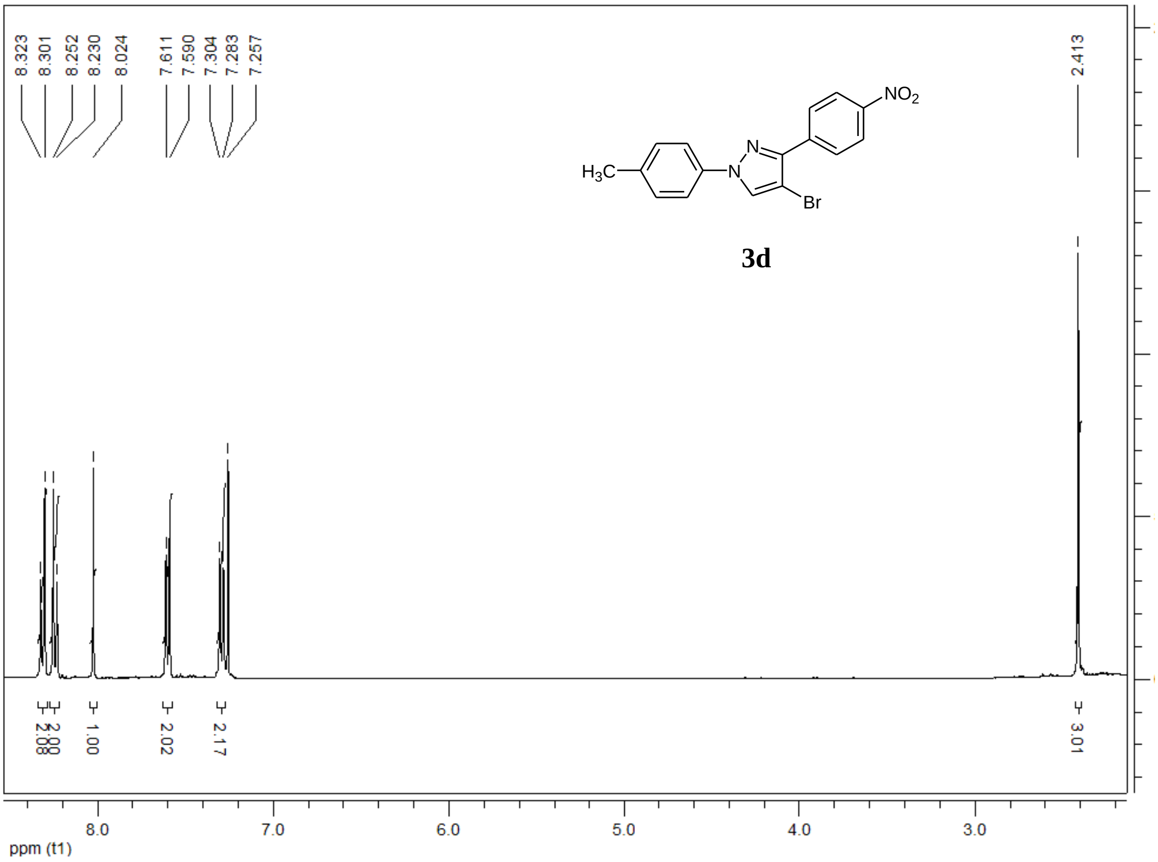
8.323
8.301
8.252
8.230
8.024
7.611
7.590
7.304
7.283
7.257
2.413
NO_2
N
H_3C
N
Br
3d
2.08
2.00
1.00
2.02
2.17
3.01
8.0
7.0
6.0
5.0
4.0
3.0
ppm (t1)

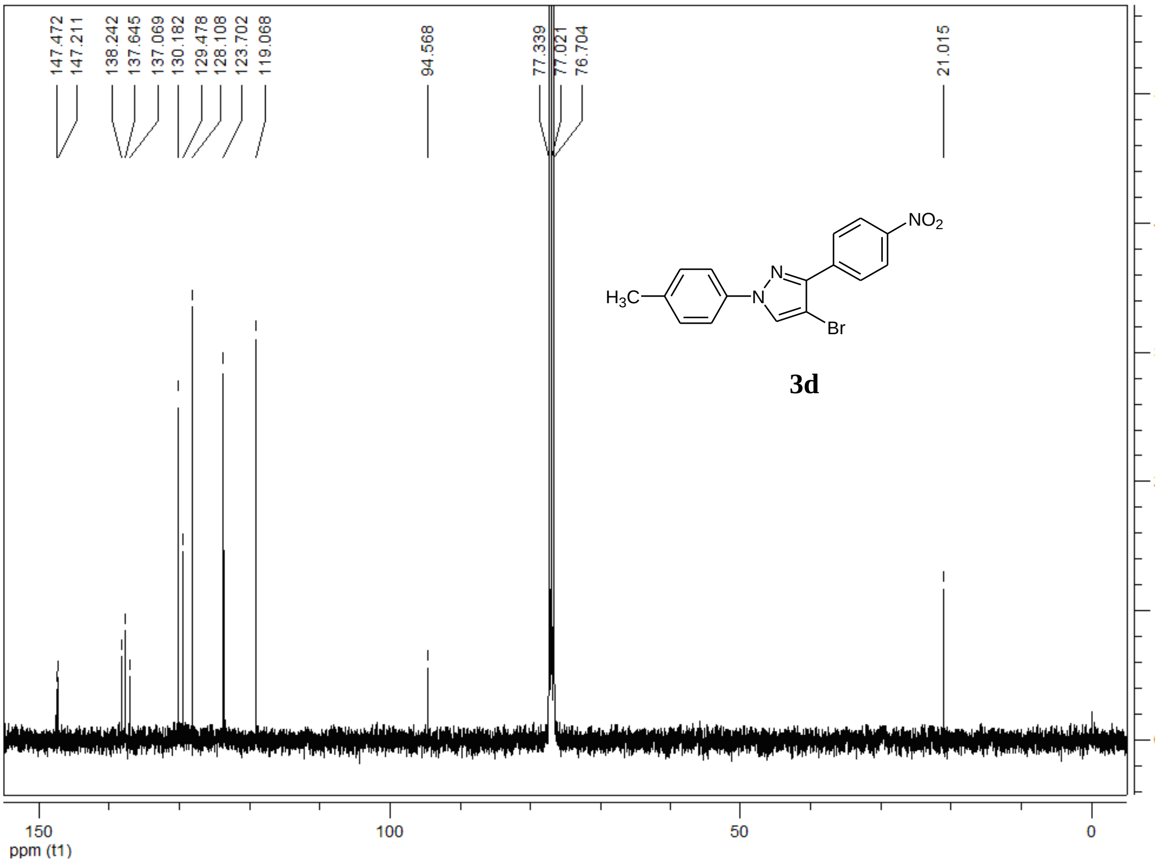
147.472
147.211
138.242
137.645
137.069
130.182
129.478
128.108
123.702
119.068
94.568
77.339
77.021
76.704
21.015
NO_2
N
H_3C
N
Br
3d
150
100
50
0
ppm (t1)

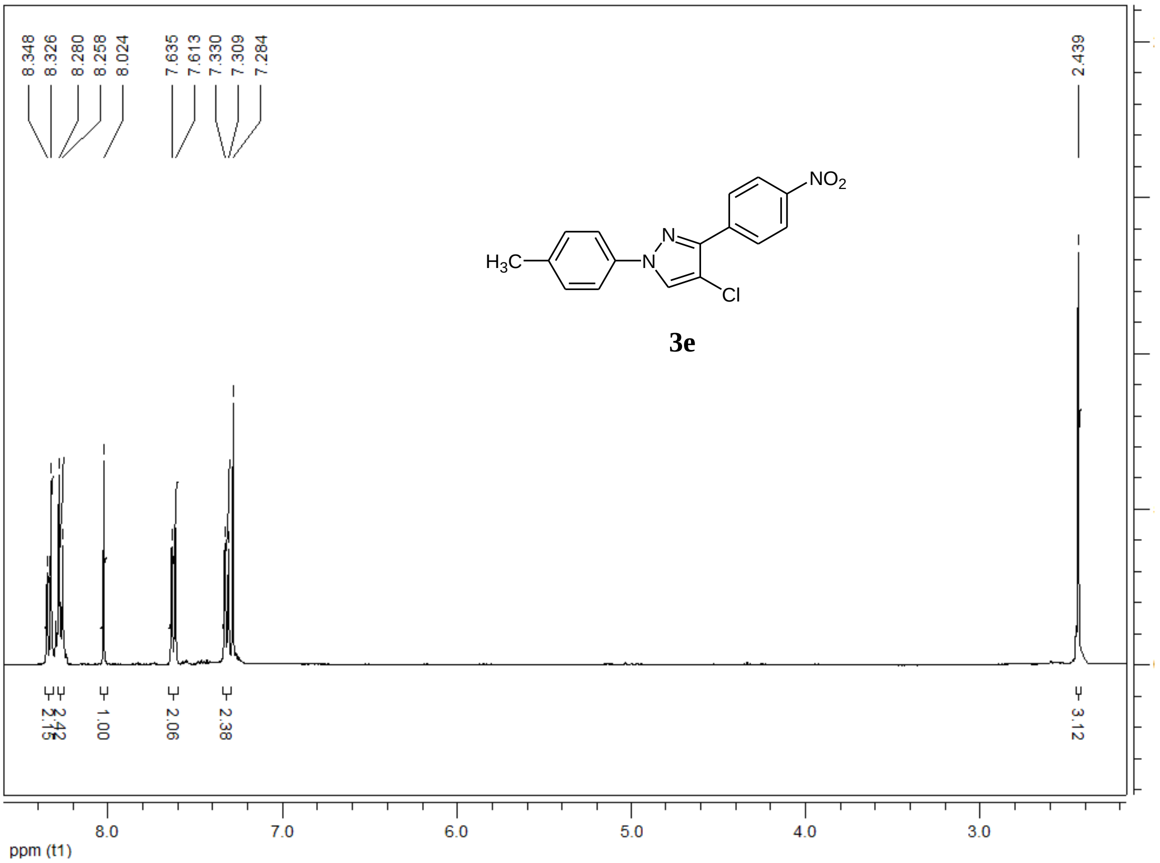
8.348
8.326
8.280
8.258
8.024
7.635
7.613
7.330
7.309
7.284
2.439
NO2
N
H3C
N
Cl
3e
2.15
2.42
1.00
2.06
2.38
3.12
8.0
7.0
6.0
5.0
4.0
3.0
ppm (t1)

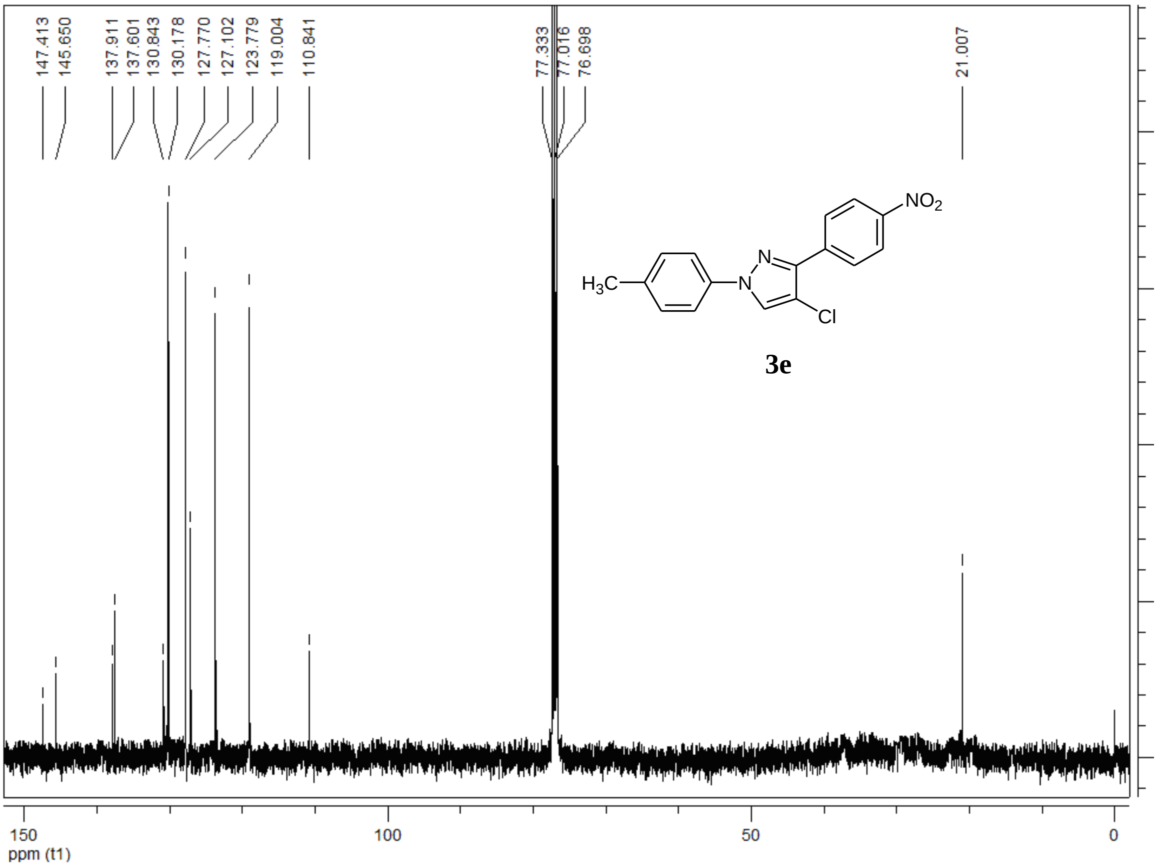
147.413
145.650
137.911
137.601
130.843
130.178
127.770
127.102
123.779
119.004
110.841
77.333
77.016
76.698
21.007
NO2
N
H3C
N
Cl
3e
150
100
50
0
ppm (t1)

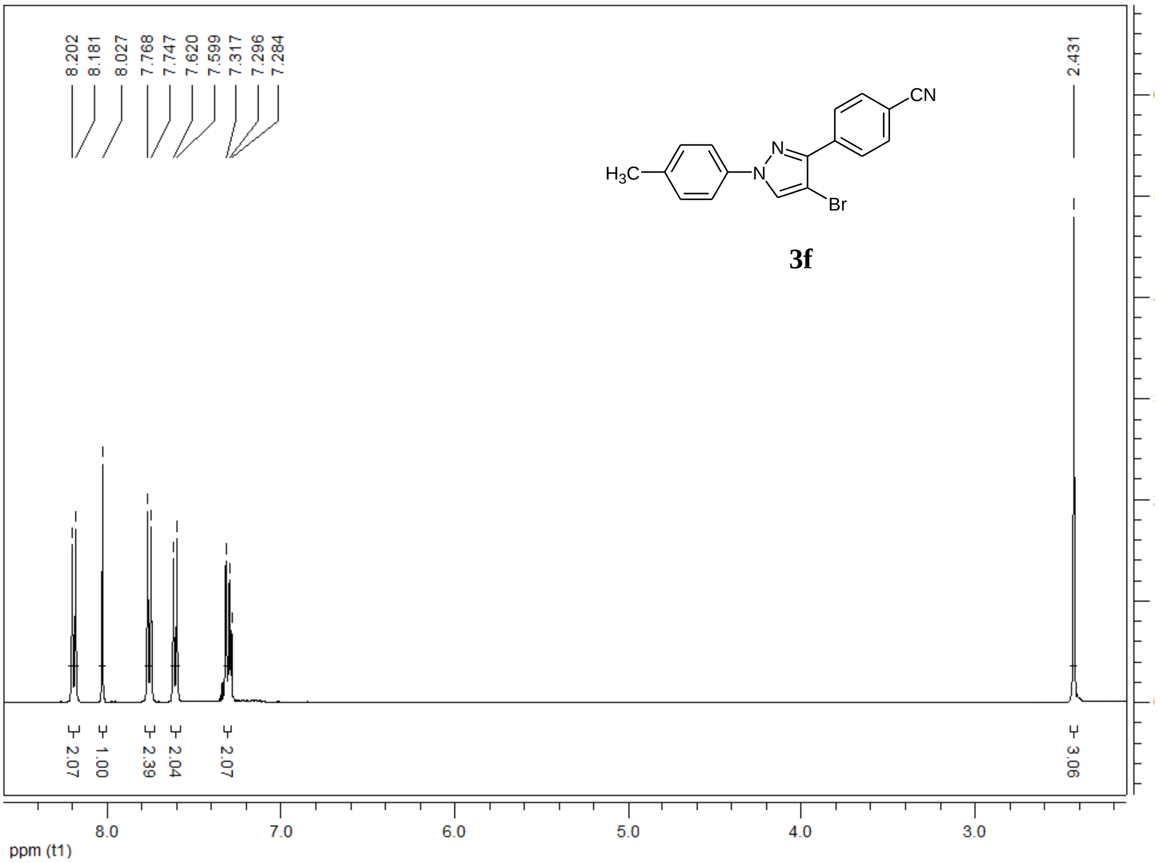
8.202
8.181
8.027
7.768
7.747
7.620
7.599
7.317
7.296
7.284
2.431
CN
N
H3C
N
Br
3f
2.07
1.00
2.39
2.04
2.07
3.06
8.0
7.0
6.0
5.0
4.0
3.0
ppm (t1)

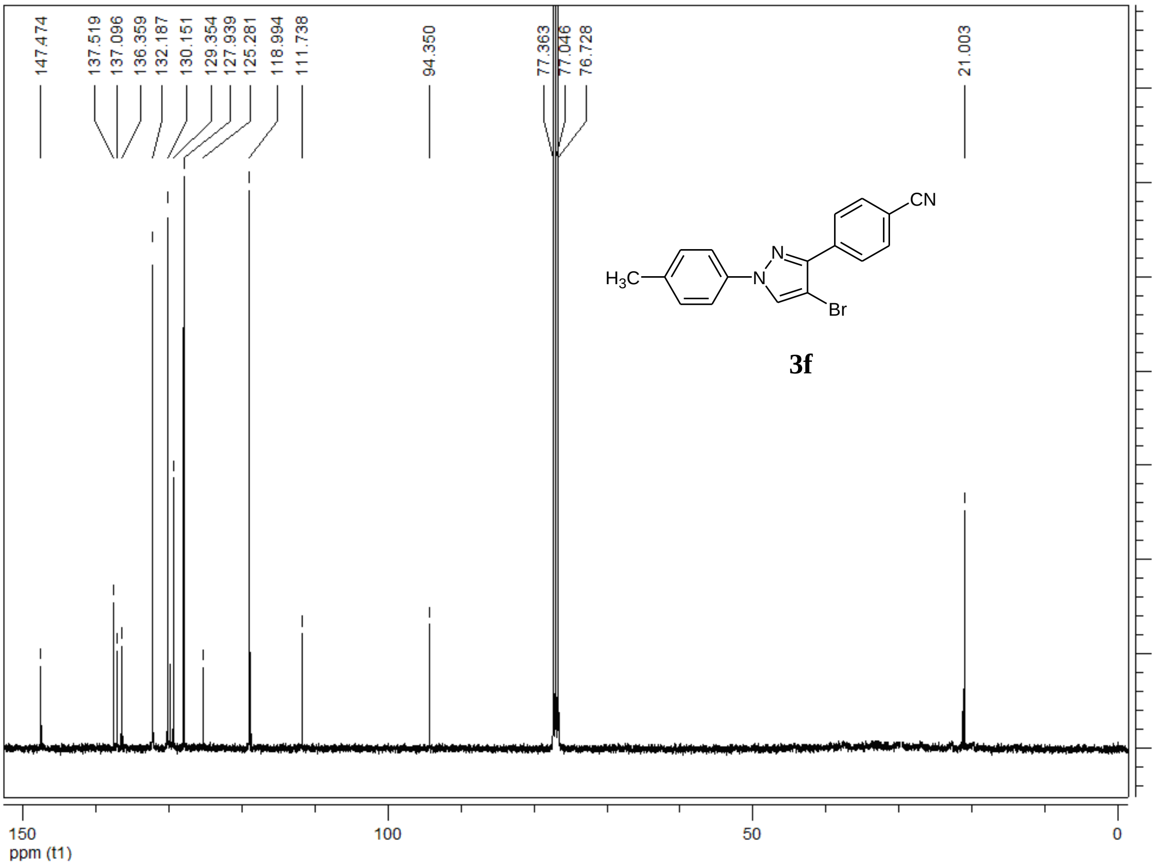
147.474
137.519
137.096
136.359
132.187
130.151
129.354
127.939
125.281
118.994
111.738
94.350
77.363
77.046
76.728
21.003
CN
N
H3C
N
Br
3f
150
100
50
0
ppm (t1)

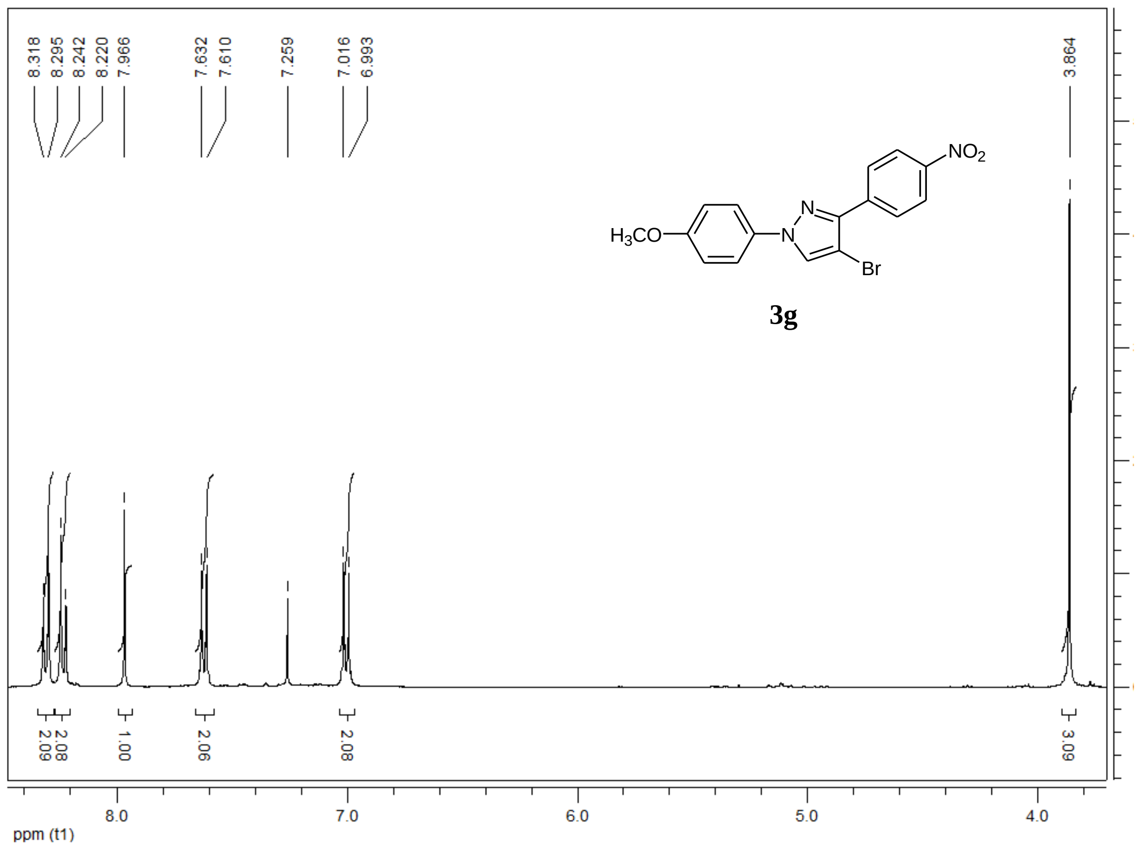
8.318
8.295
8.242
8.220
7.966
7.632
7.610
7.259
7.016
6.993
3.864
H_3CO
N
N
NO_2
Br
2.09
2.08
1.00
2.06
2.08
3.09
8.0
7.0
6.0
5.0
4.0
ppm (t1)

3g

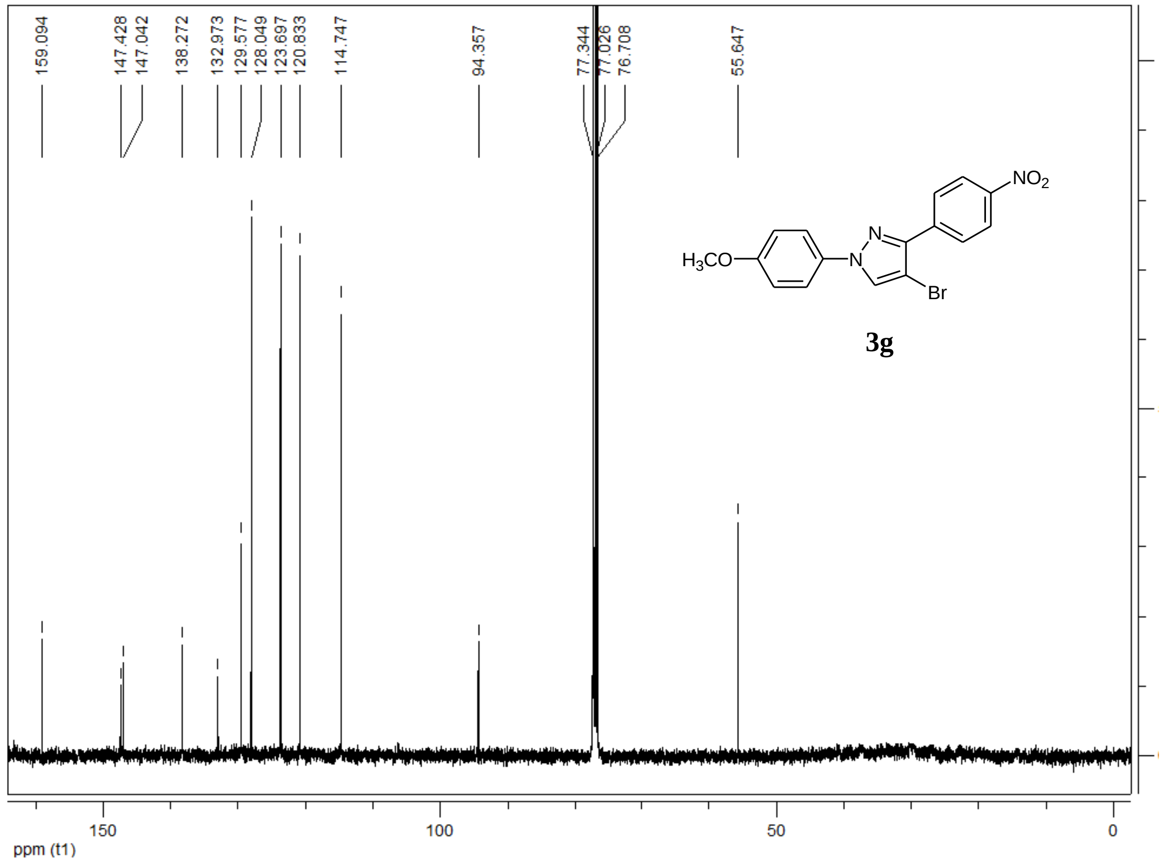
159.094
147.428
147.042
138.272
132.973
129.577
128.049
123.697
120.833
114.747
94.357
77.344
77.026
76.708
55.647
H_3CO
N
N
NO_2
Br
150
100
50
0
ppm (t1)

3g

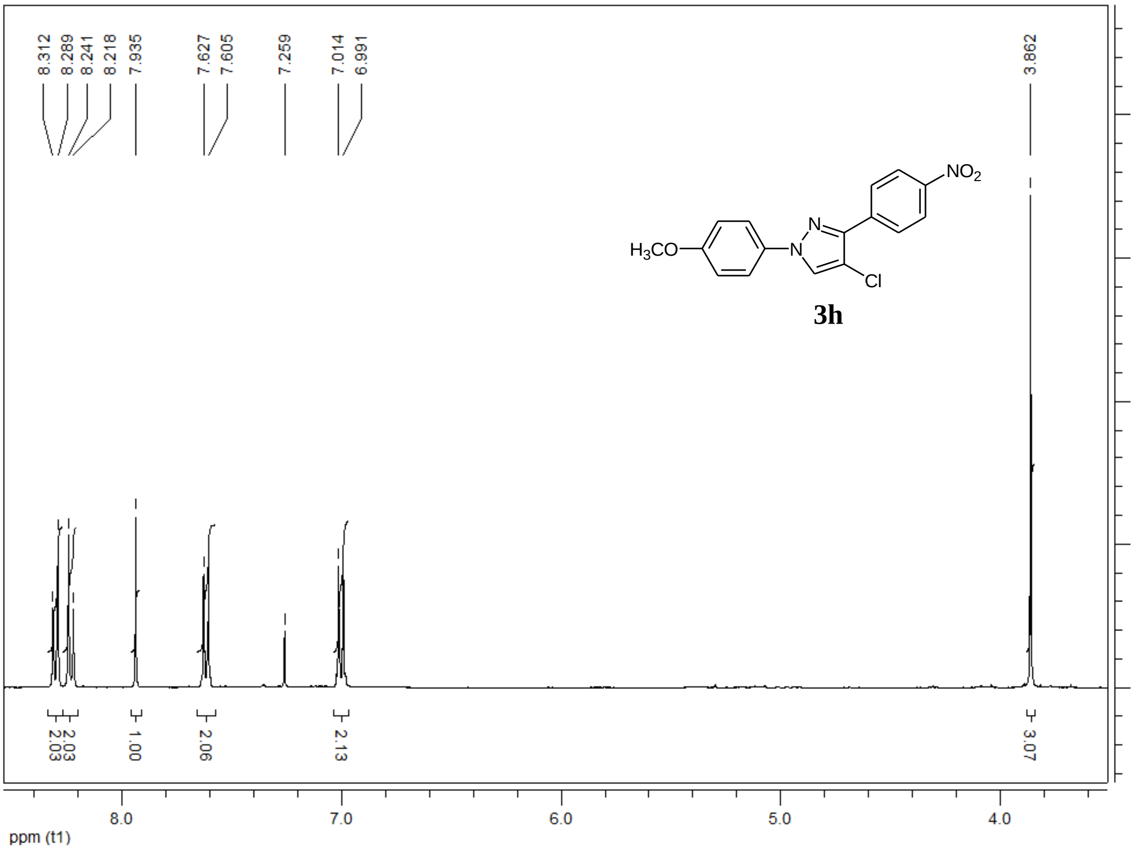
8.312
8.289
8.241
8.218
7.935
7.627
7.605
7.259
7.014
6.991
3.862
H_3CO
N
N
NO_2
Cl
2.03
2.03
1.00
2.06
2.13
3.07
8.0
7.0
6.0
5.0
4.0
ppm (t1)

3h

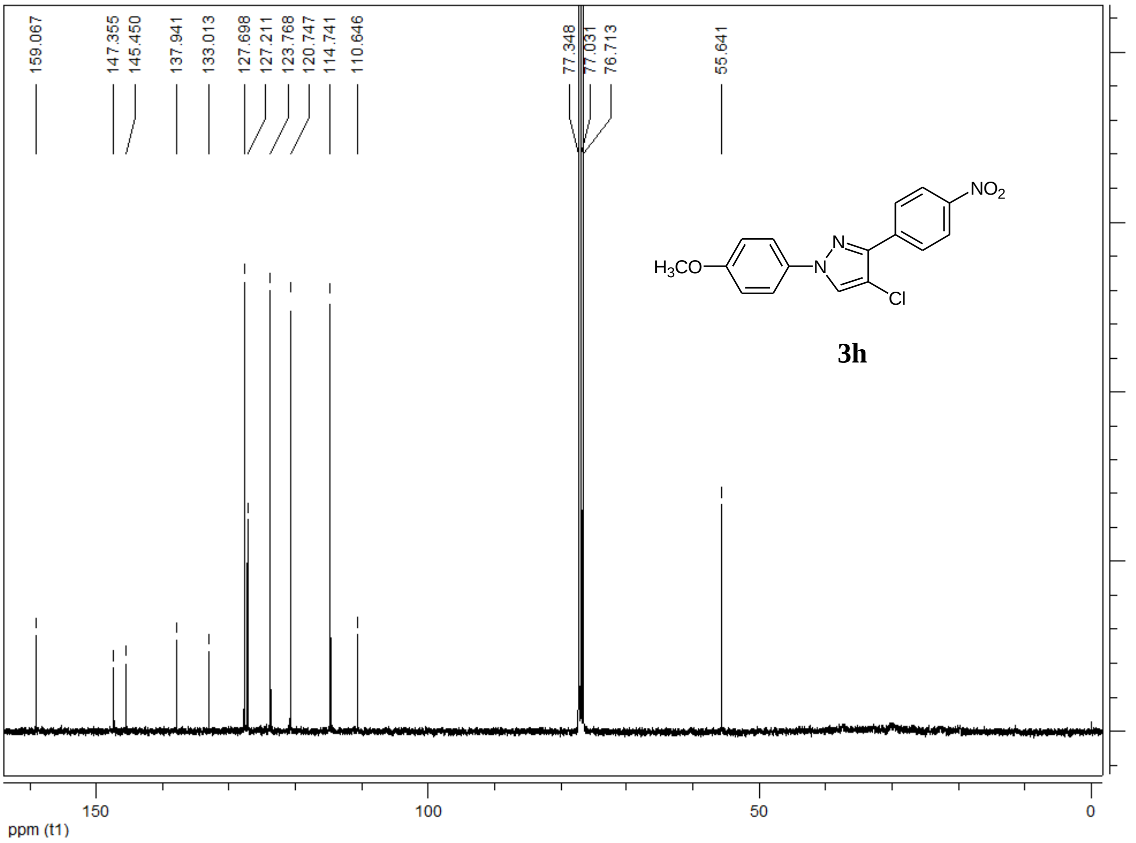
159.067
147.355
145.450
137.941
133.013
127.698
127.211
123.768
120.747
114.741
110.646
77.348
77.031
76.713
55.641
H_3CO
N
N
NO_2
Cl
150
100
50
0
ppm (t1)

3h

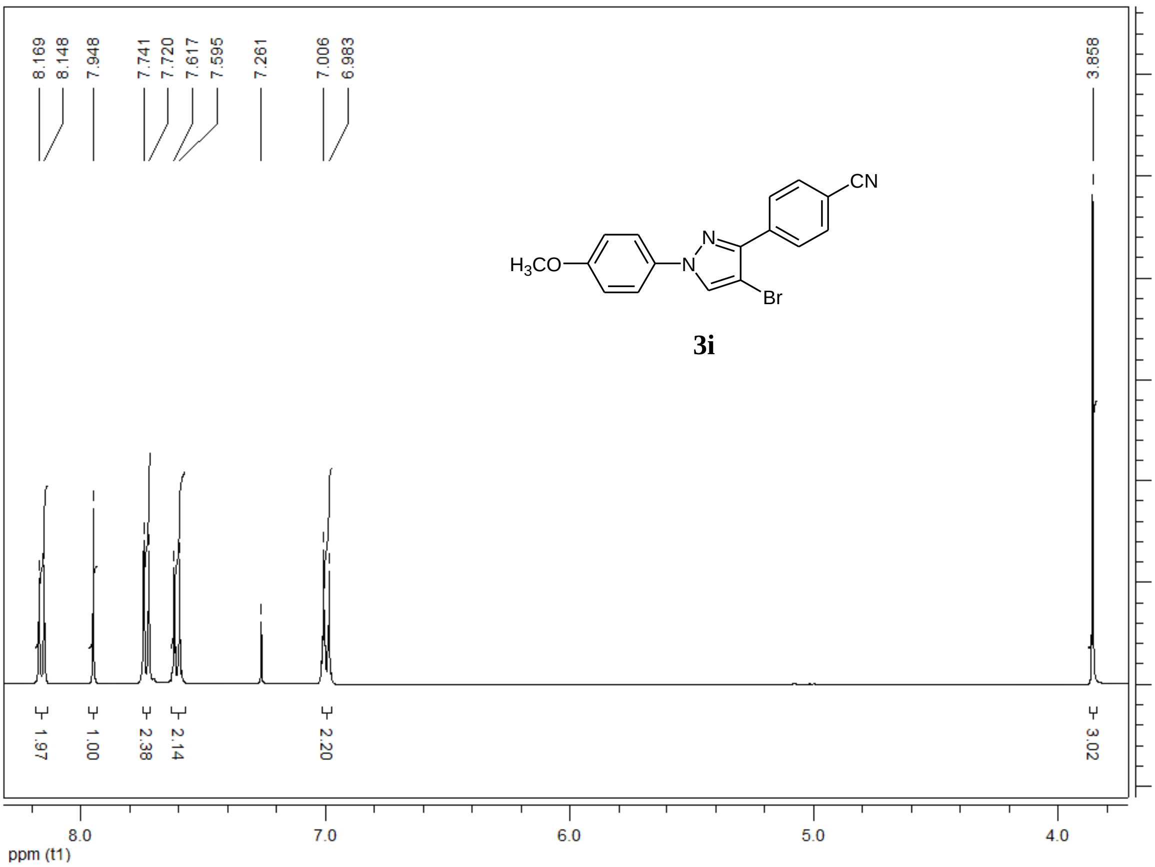
8.169
8.148
7.948
7.741
7.720
7.617
7.595
7.261
7.006
6.983
3.858
CN
N
H3CO
N
Br
3i
1.97
1.00
2.38
2.14
2.20
3.02
8.0
7.0
6.0
5.0
4.0
ppm (t1)

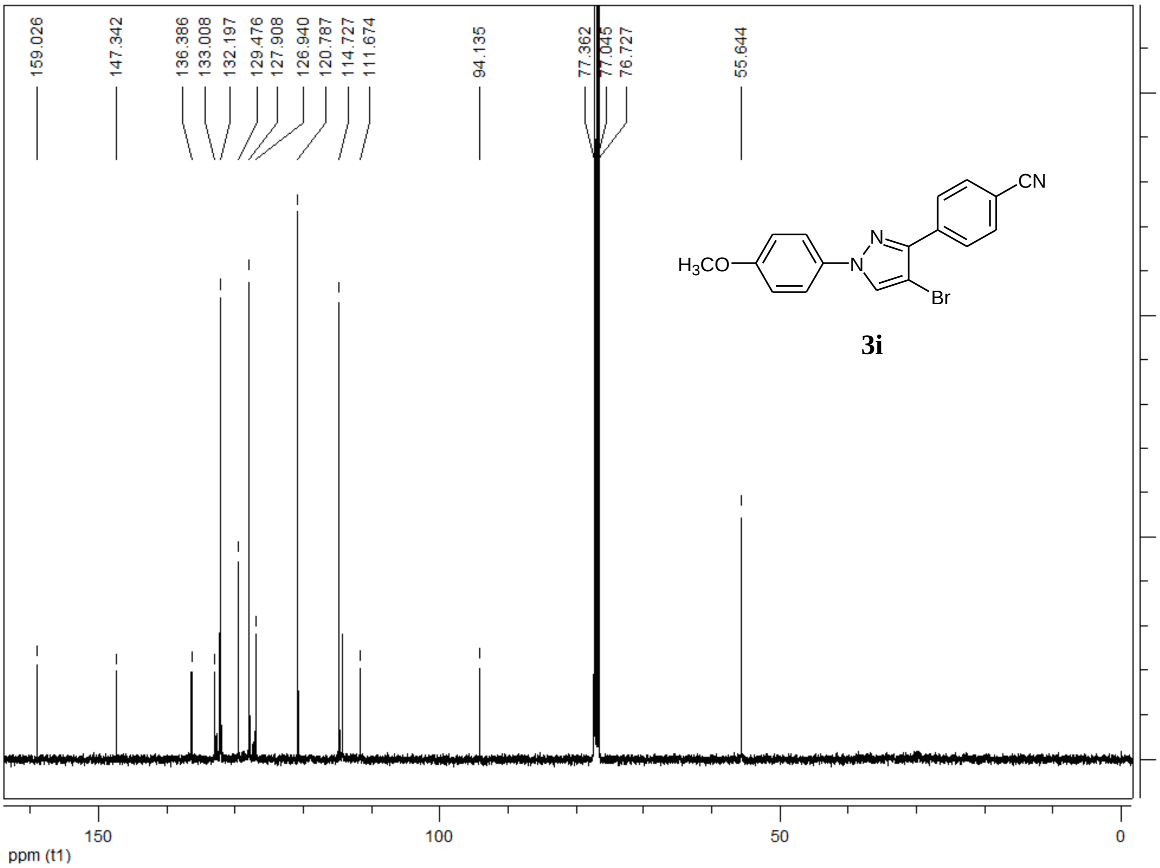
159.026
147.342
136.386
133.008
132.197
129.476
127.908
126.940
120.787
114.727
111.674
94.135
77.362
77.045
76.727
55.644
CN
N
H3CO
N
Br
3i
150
100
50
0
ppm (t1)

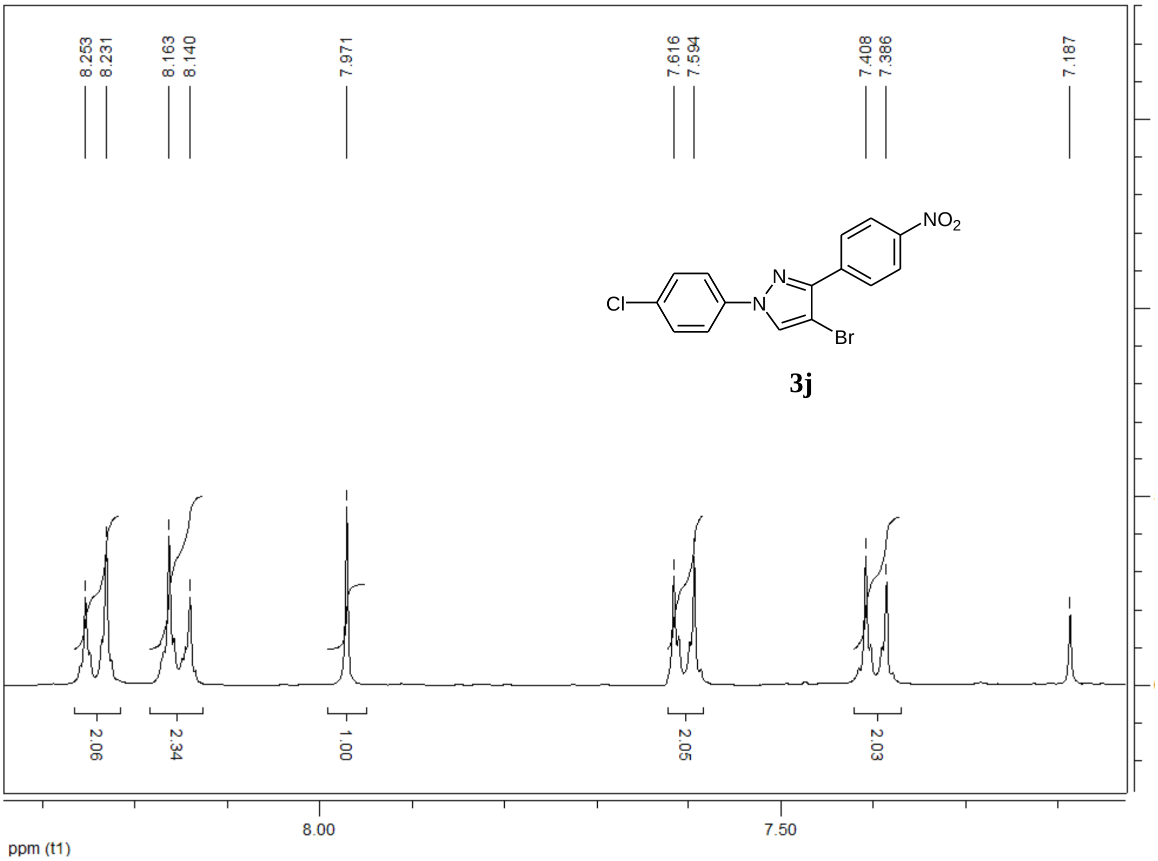
8.253
8.231
8.163
8.140
7.971
7.616
7.594
7.408
7.386
7.187
NO2
Cl
N
N
Br
3j
2.06
2.34
1.00
2.05
2.03
8.00
7.50
ppm (t1)

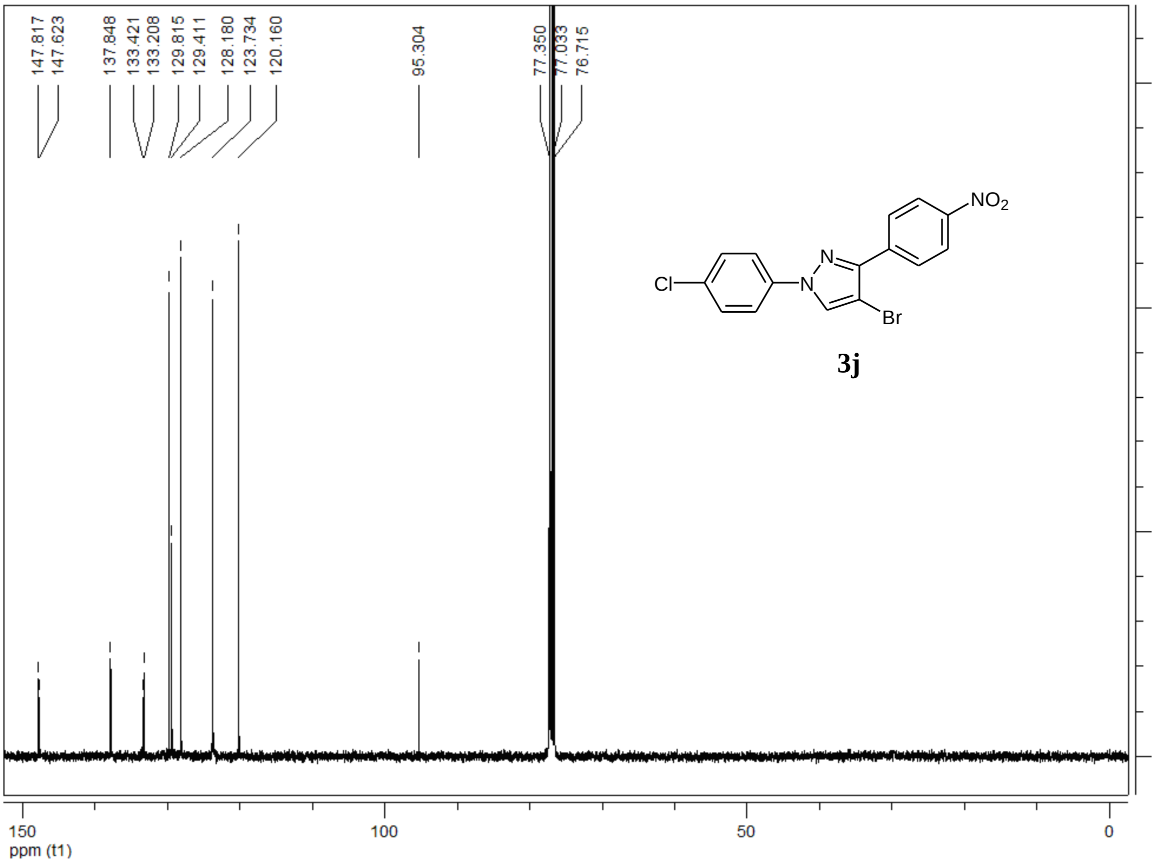
147.817
147.623
137.848
133.421
133.208
129.815
129.411
128.180
123.734
120.160
95.304
77.350
77.033
76.715
NO2
Cl
N
N
Br
3j
150
100
50
0
ppm (t1)

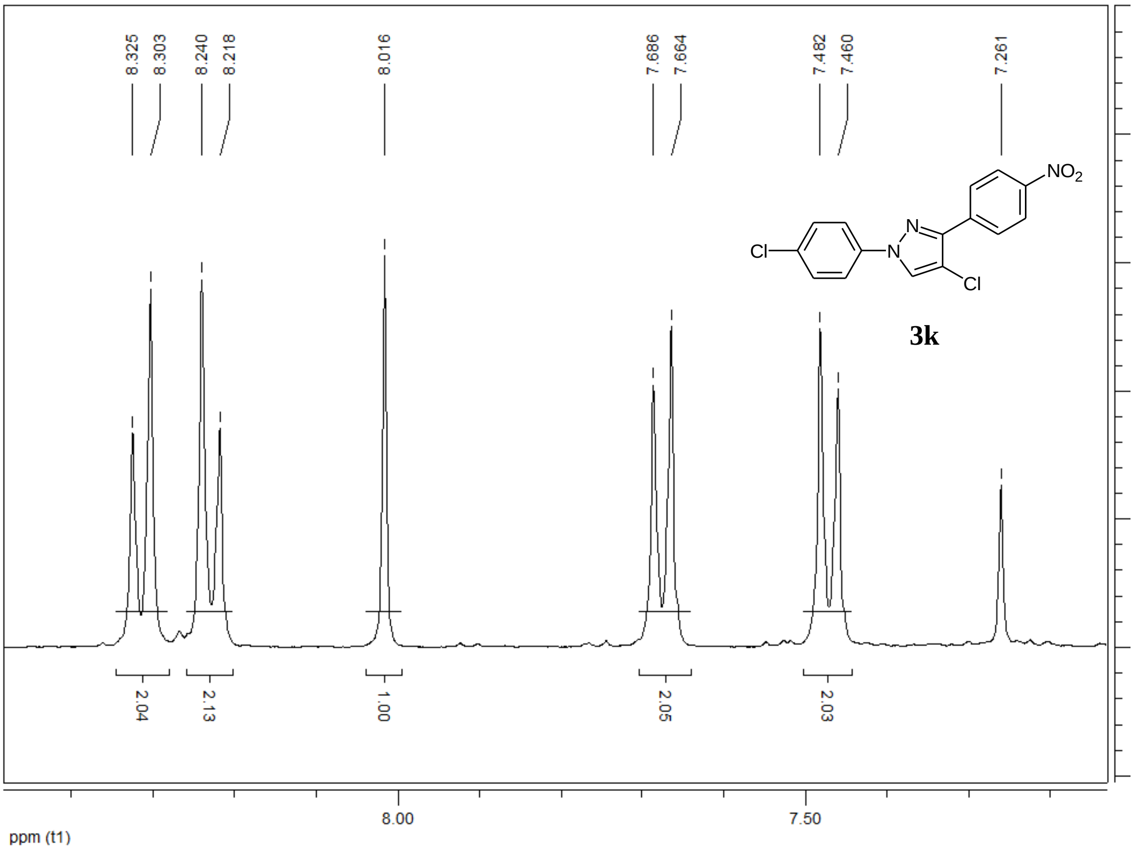
8.325
8.303
8.240
8.218
8.016
7.686
7.664
7.482
7.460
7.261
NO2
Cl
N
N
Cl
2.04
2.13
1.00
2.05
2.03
8.00
7.50
ppm (t1)

3k

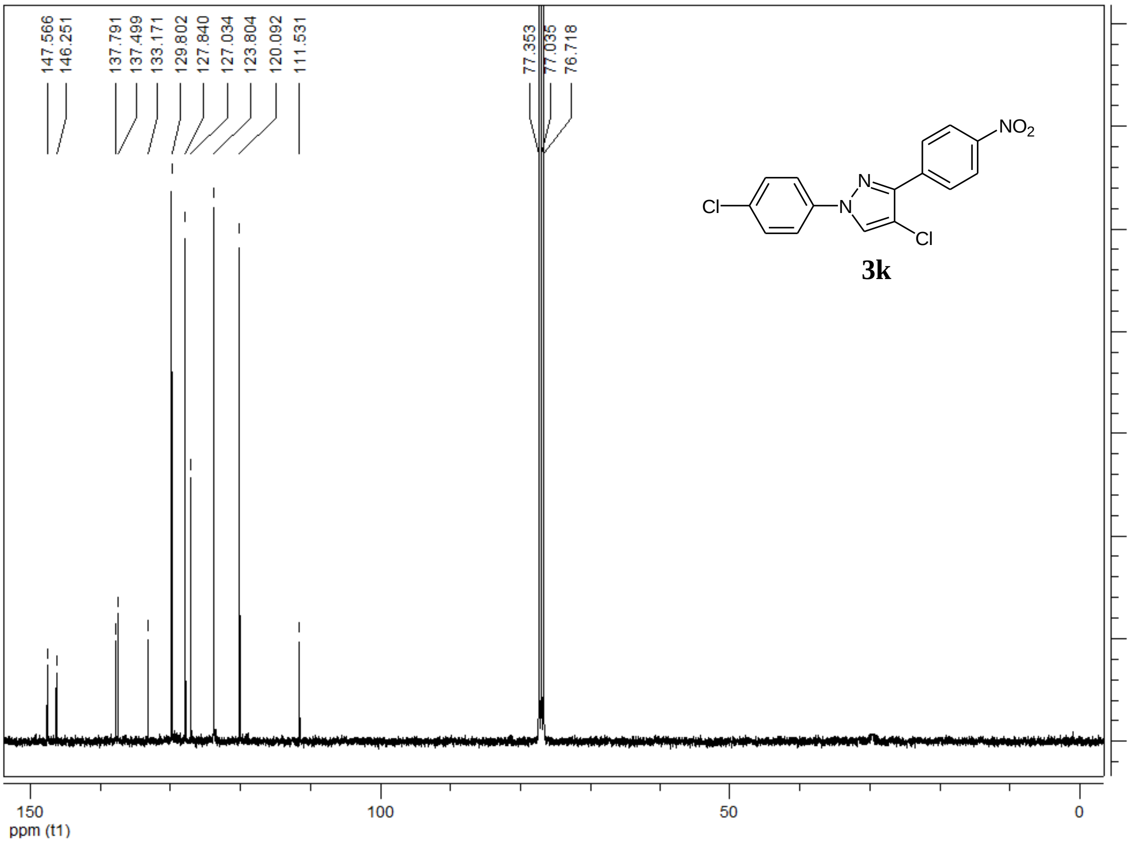
147.566
146.251
137.791
137.499
133.171
129.802
127.840
127.034
123.804
120.092
111.531
77.353
77.035
76.718
NO2
Cl
N
N
Cl
150
100
50
0
ppm (t1)

3k

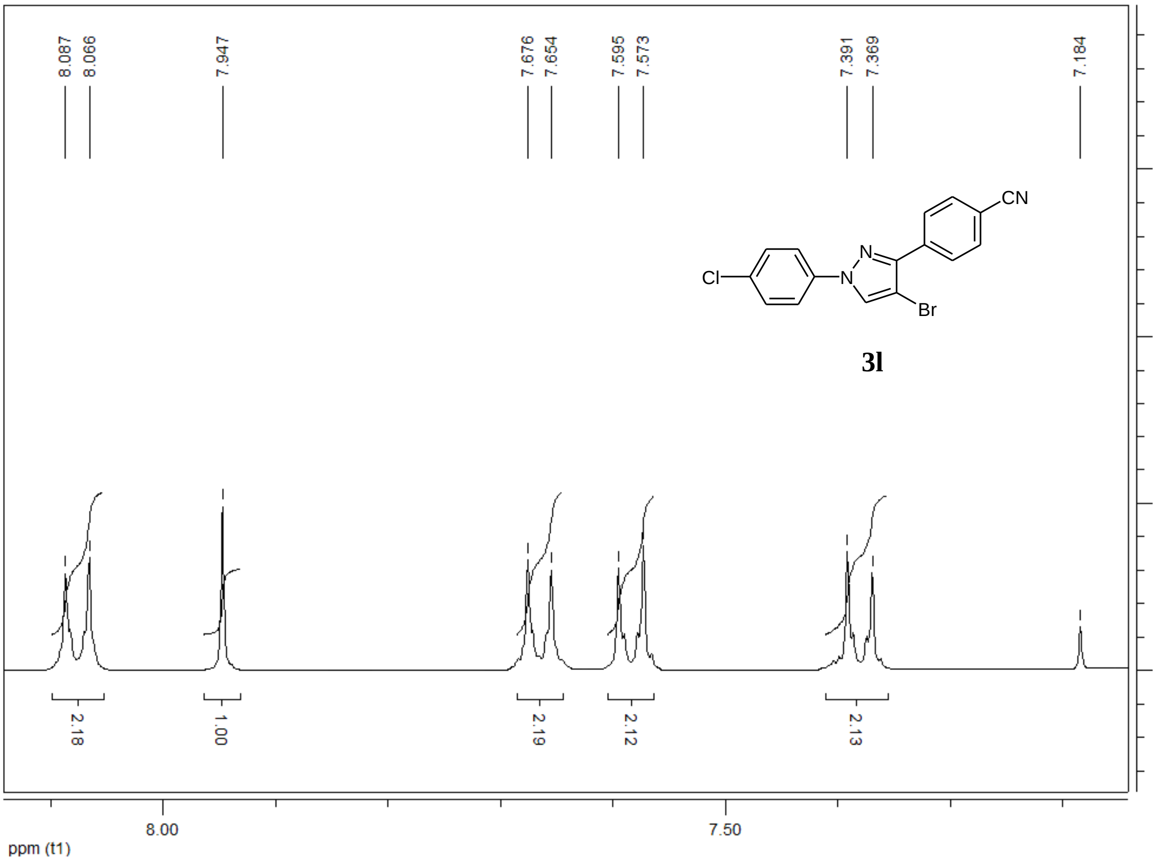
8.087
8.066
7.947
7.676
7.654
7.595
7.573
7.391
7.369
7.184
CN
N
Cl
N
Br
2.18
1.00
2.19
2.12
2.13
8.00
7.50
ppm (t1)

3l

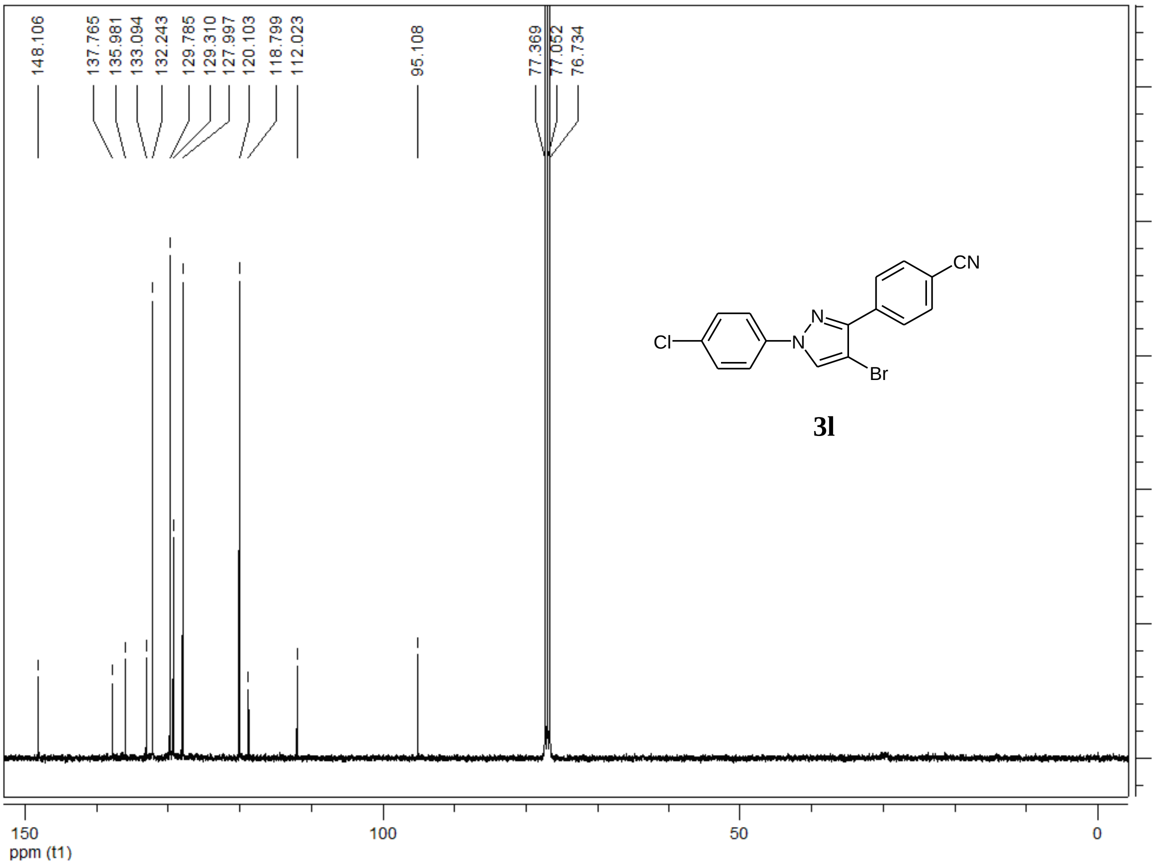
148.106
137.765
135.981
133.094
132.243
129.785
129.310
127.997
120.103
118.799
112.023
95.108
77.369
77.052
76.734
CN
N
Cl
N
Br
150
100
50
0
ppm (t1)

3l

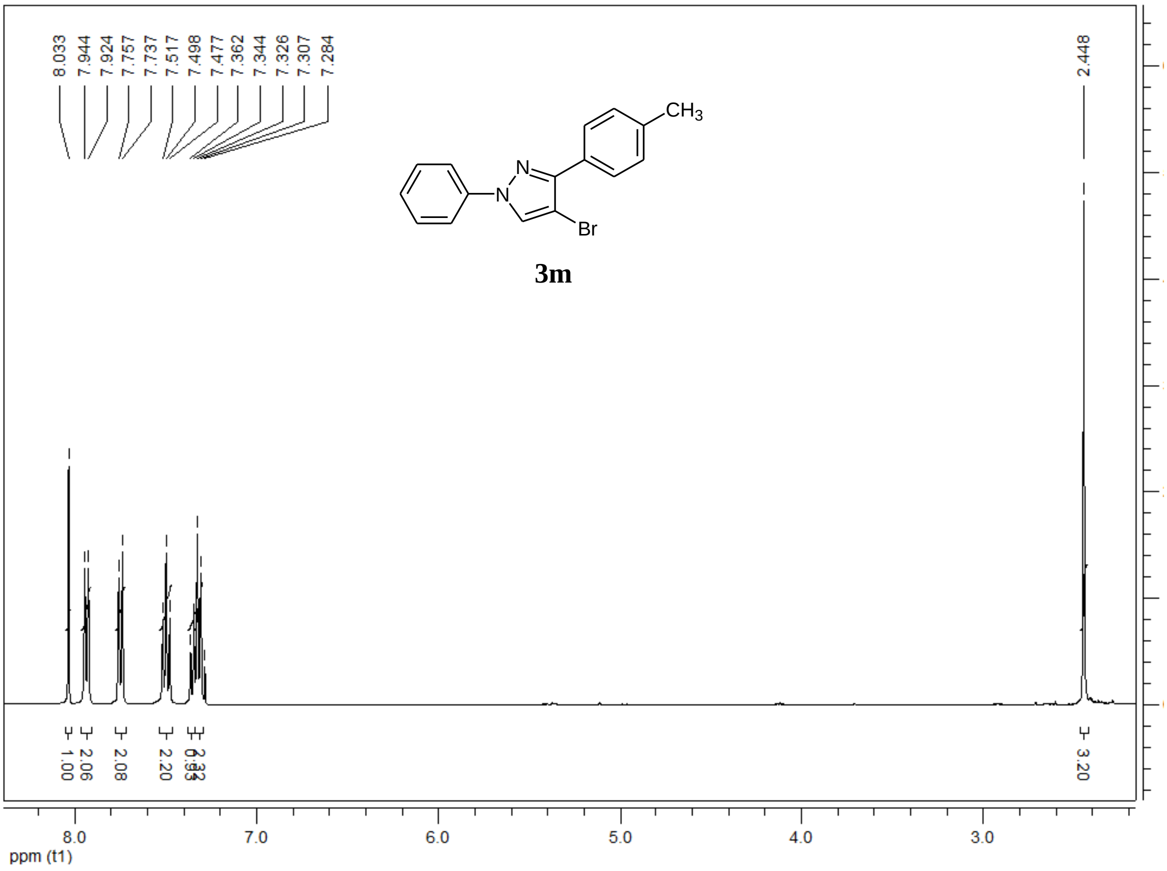
8.033
7.944
7.924
7.757
7.737
7.517
7.498
7.477
7.362
7.344
7.326
7.307
7.284
2.448
CH3
N
N
Br
3m
1.00
2.06
2.08
2.20
3.20
8.0
7.0
6.0
5.0
4.0
3.0
ppm (t1)

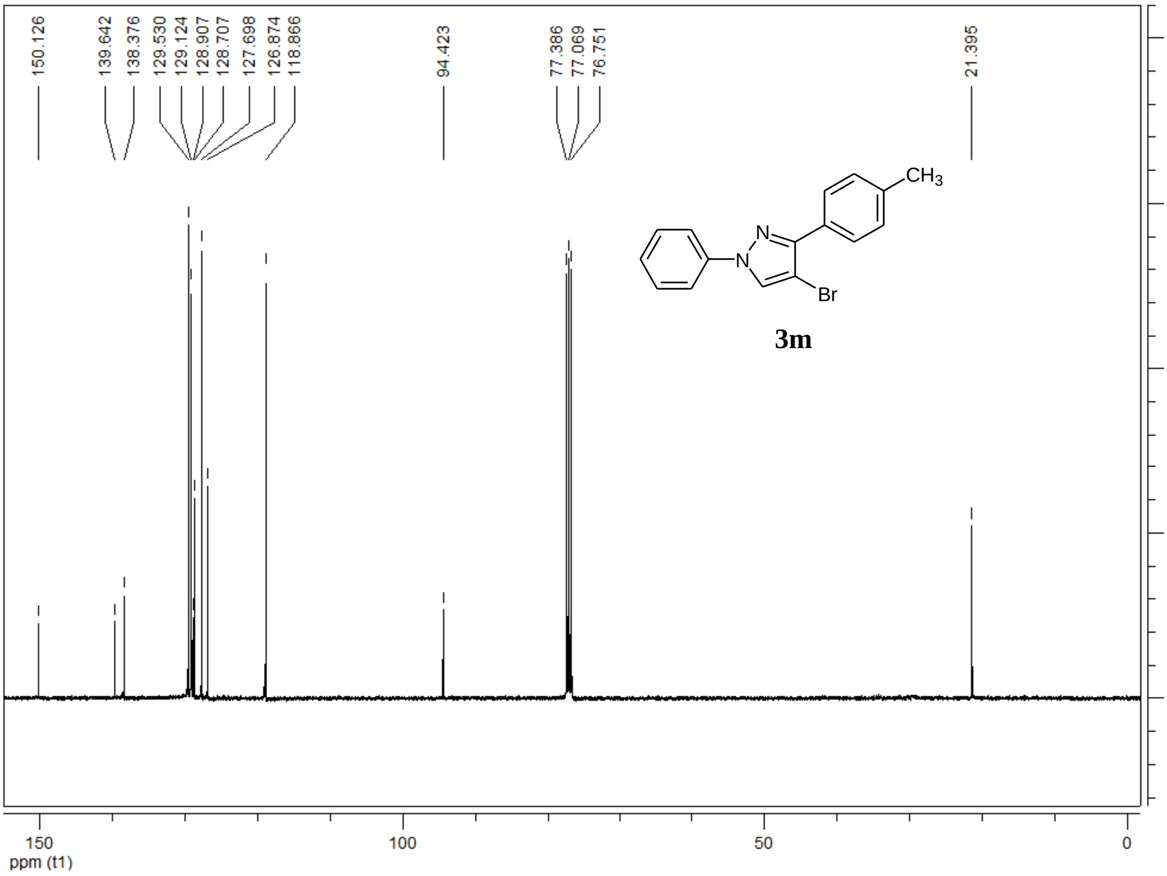
150.126
139.642
138.376
129.530
129.124
128.907
128.707
127.698
126.874
118.866
94.423
77.386
77.069
76.751
21.395
CH3
N
N
Br
3m
150
100
50
0
ppm (t1)

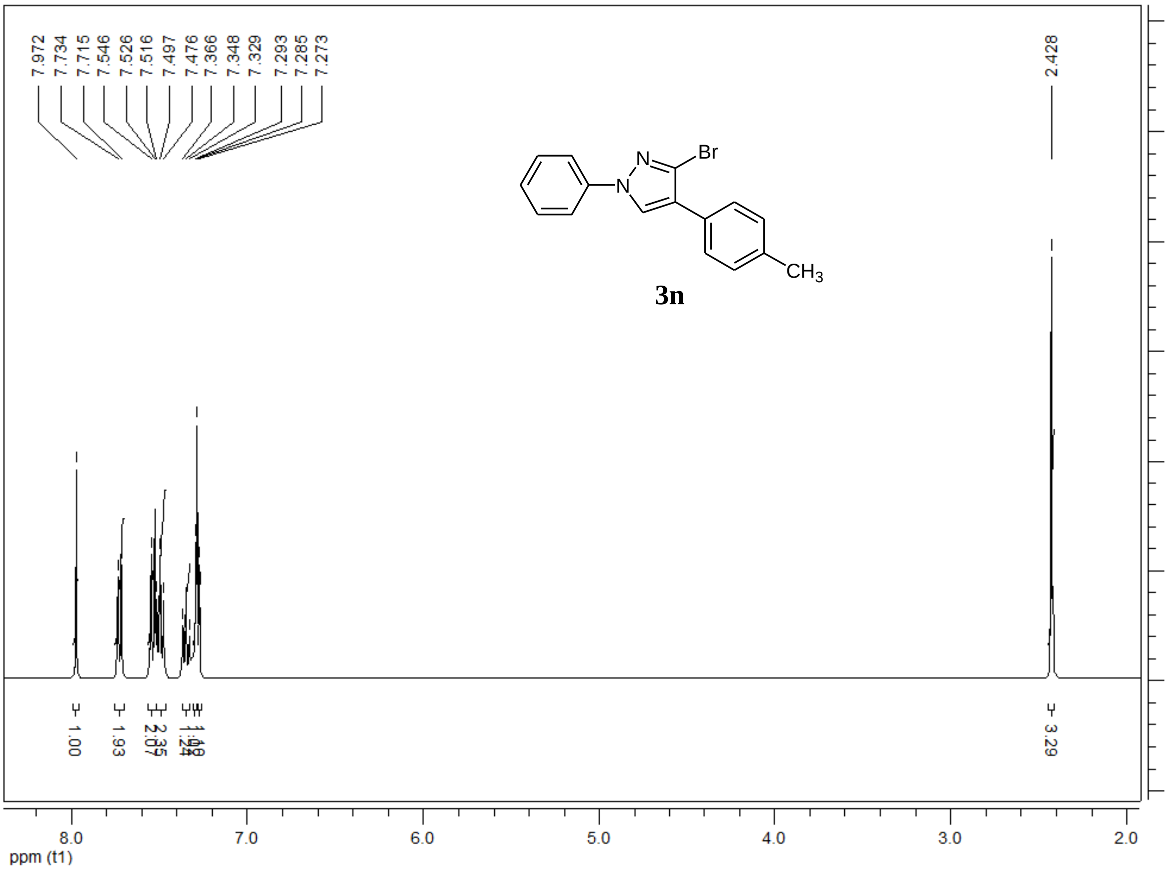
7.972
7.734
7.715
7.546
7.526
7.516
7.497
7.476
7.366
7.348
7.329
7.293
7.285
7.273
2.428
Br
N
N
CH3
3n
1.00
1.93
3.29
8.0
7.0
6.0
5.0
4.0
3.0
2.0
ppm (t1)

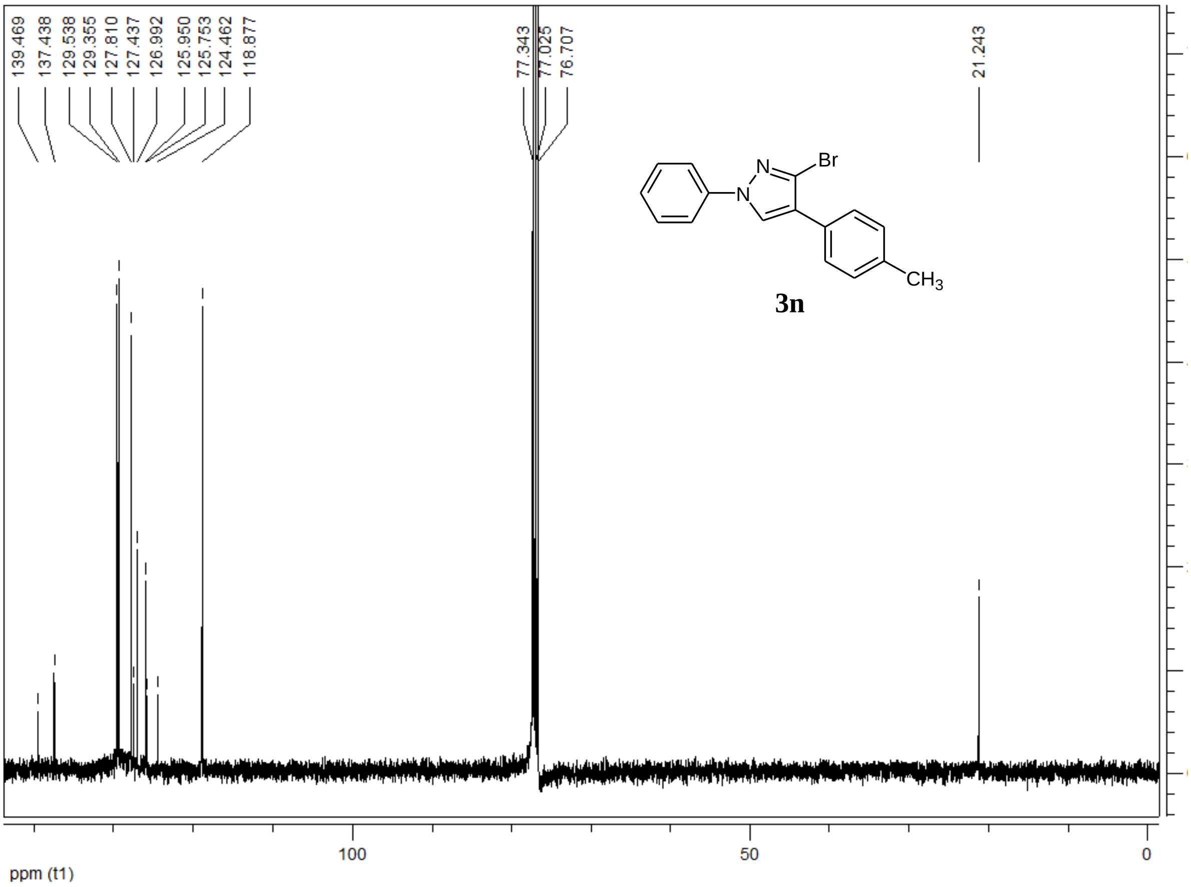
139.469
137.438
129.538
129.355
127.810
127.437
126.992
125.950
125.753
124.462
118.877
77.343
77.025
76.707
21.243
Br
N
N
CH3
3n
100
50
0
ppm (t1)

NMR spectra data for compounds 4

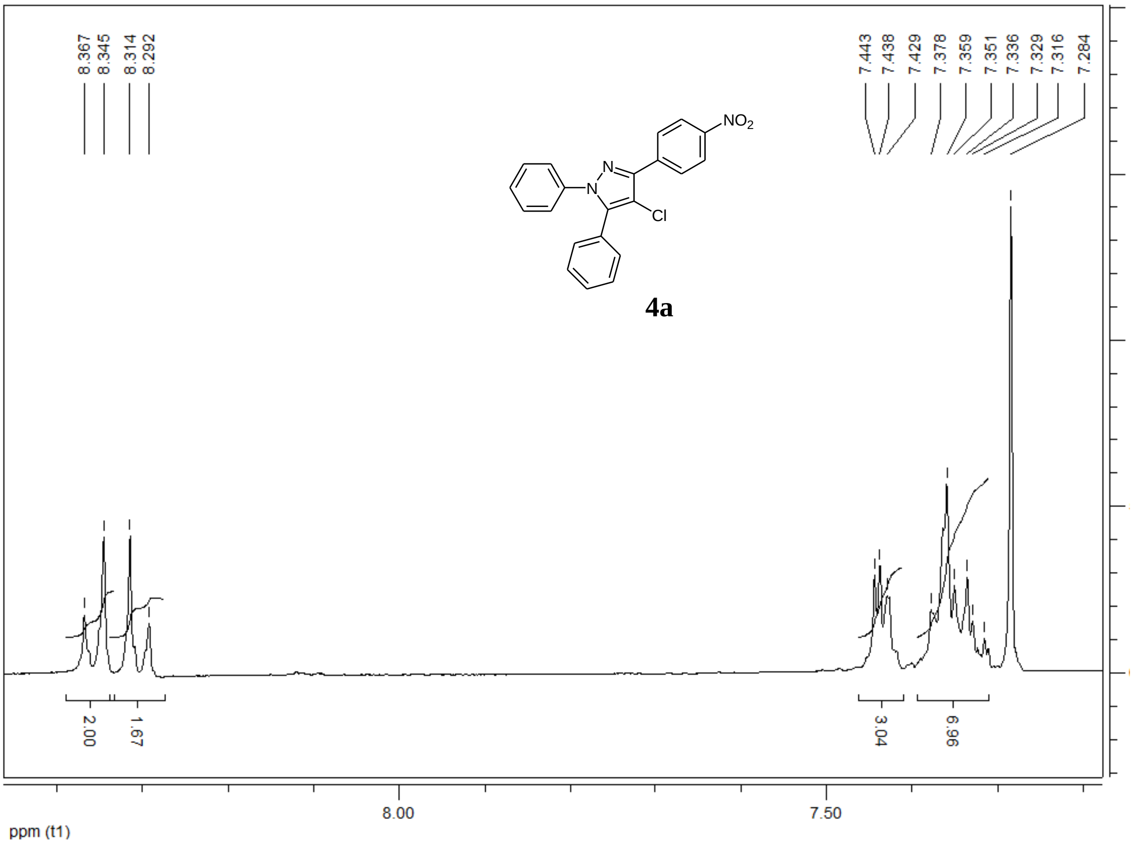

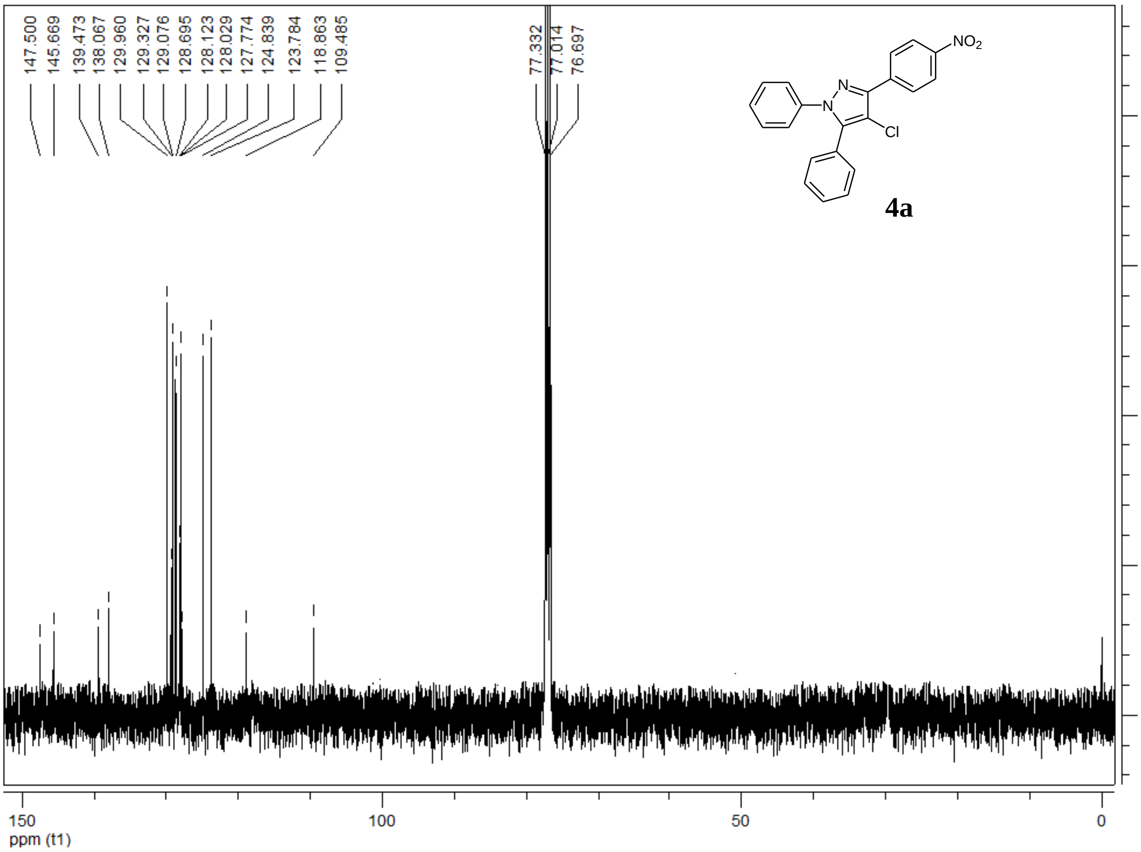

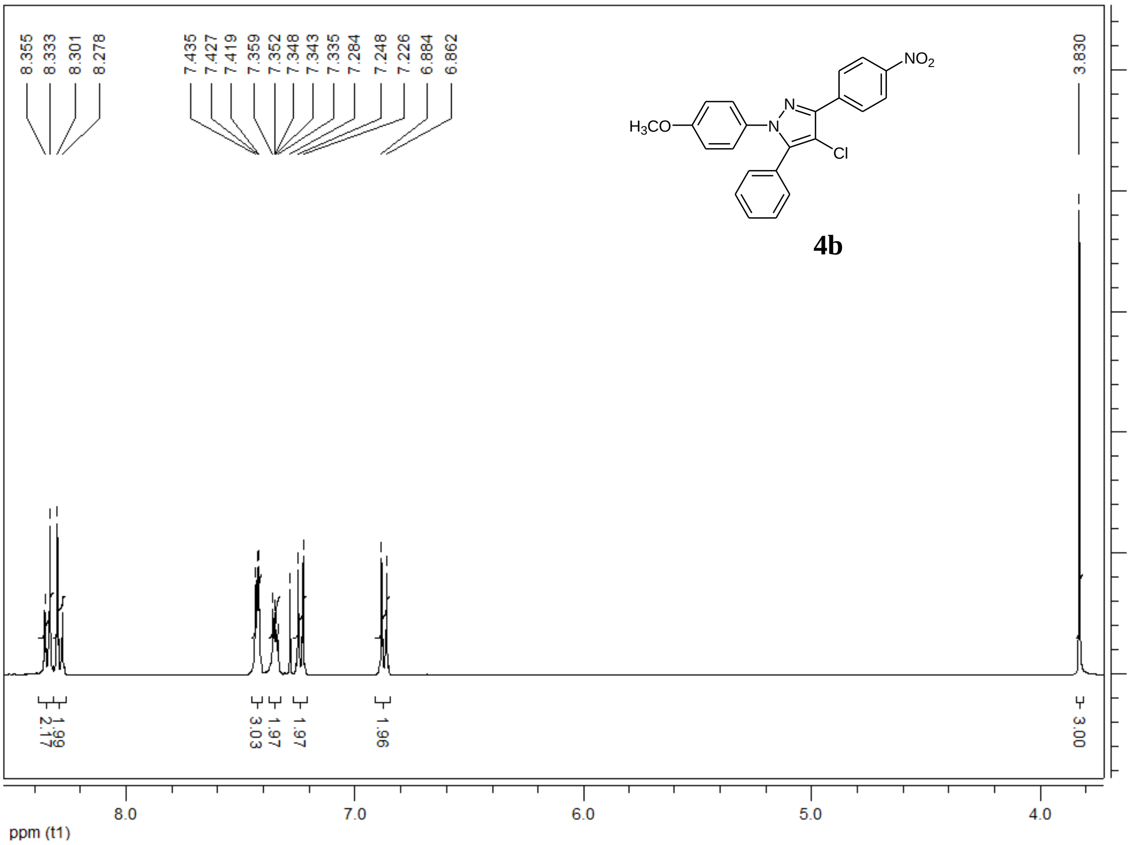
8.355
8.333
8.301
8.278
7.435
7.427
7.419
7.359
7.352
7.348
7.343
7.335
7.284
7.248
7.226
6.884
6.862
3.830
H3CO
N
N
NO2
Cl
4b
2.17
1.99
3.03
1.97
1.97
1.96
3.00
8.0
7.0
6.0
5.0
4.0
ppm (t1)

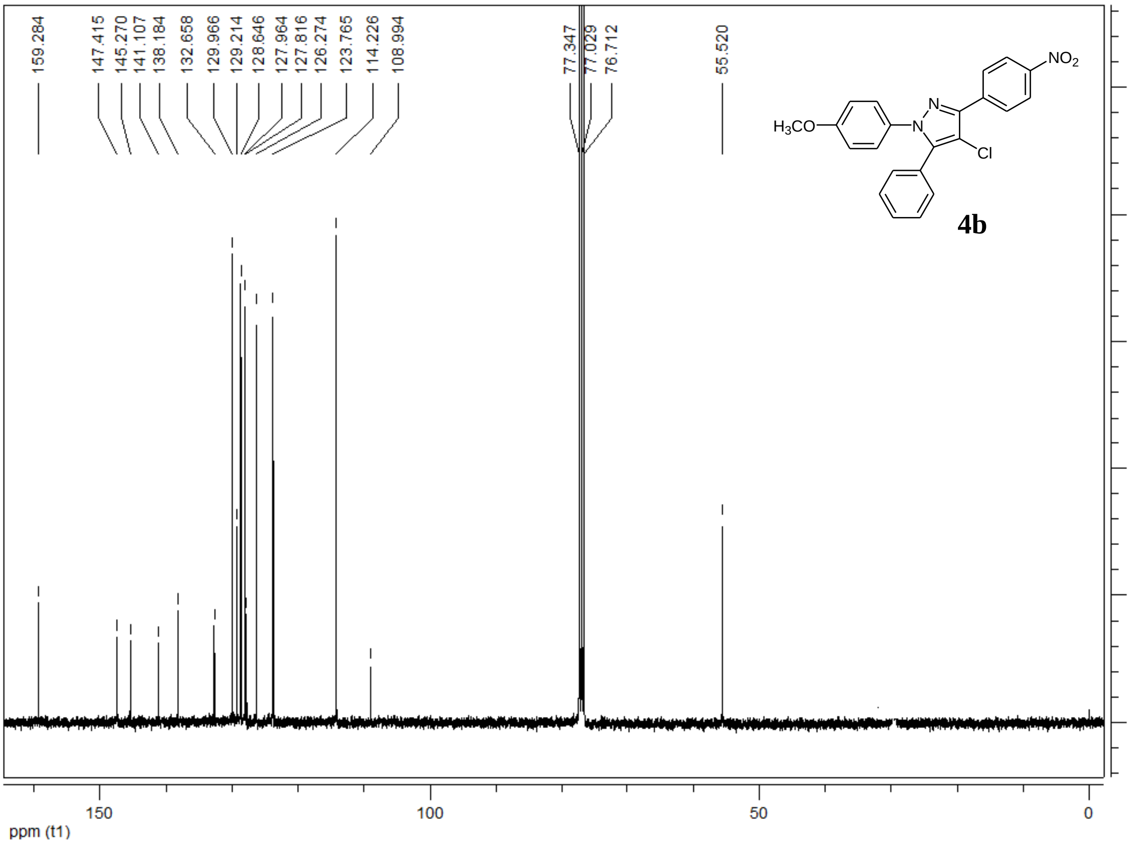
159.284
147.415
145.270
141.107
138.184
132.658
129.966
129.214
128.646
127.964
127.816
126.274
123.765
114.226
108.994
77.347
77.029
76.712
55.520
H3CO
N
N
NO2
Cl
4b
150
100
50
0
ppm (t1)

NMR spectra data for compound 5

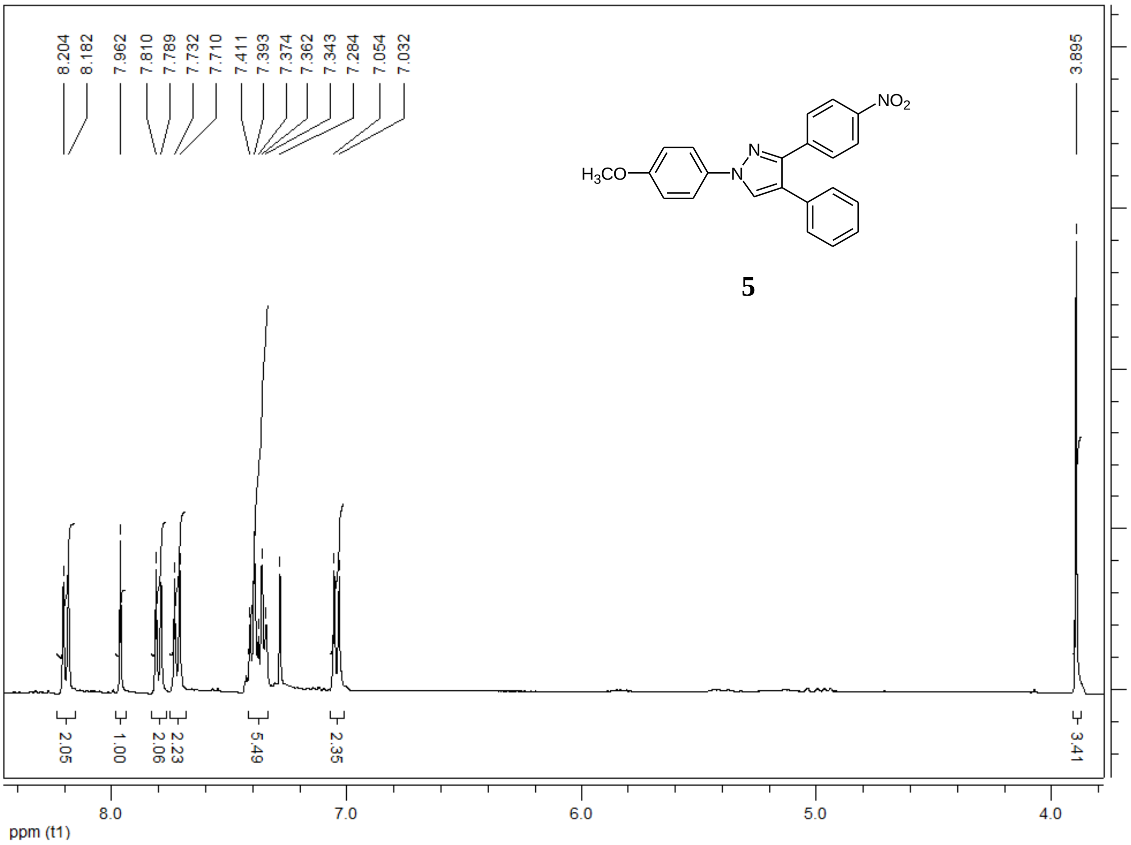

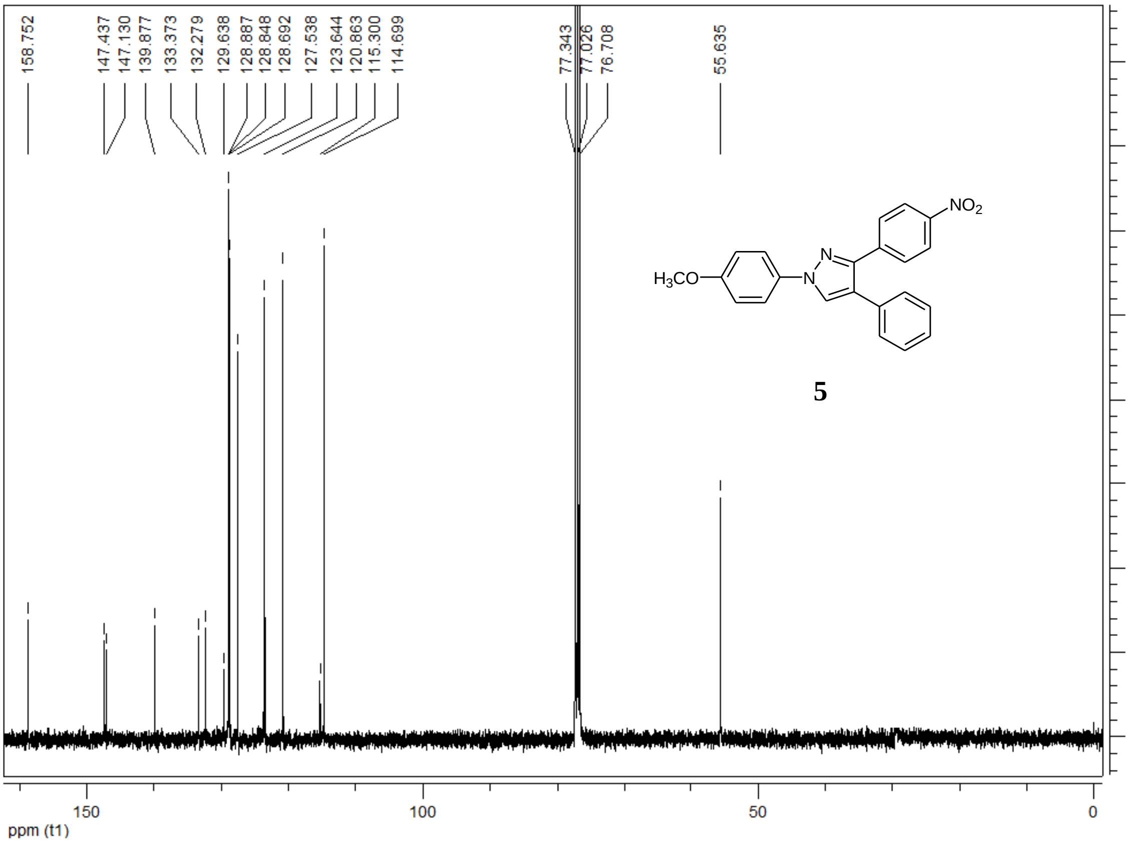

X-ray crystallography of pyrazole 3g

Single Crystal X-Ray Analysis. A representative crystal was surveyed on a Bruker APEX diffractometer. All crystallographic calculations were facilitated by the SHELXL-97 system.

_computing_data_collection	'Bruker SMART'
_computing_cell_refinement	'Bruker SMART'
_computing_data_reduction	'Bruker SAINT'
_computing_structure_solution	'SHELXS-97 (Sheldrick, 1990)'
_computing_structure_refinement	'SHELXL-97 (Sheldrick, 1997)'
_computing_molecular_graphics	XP
_computing_publication_material	XCIF

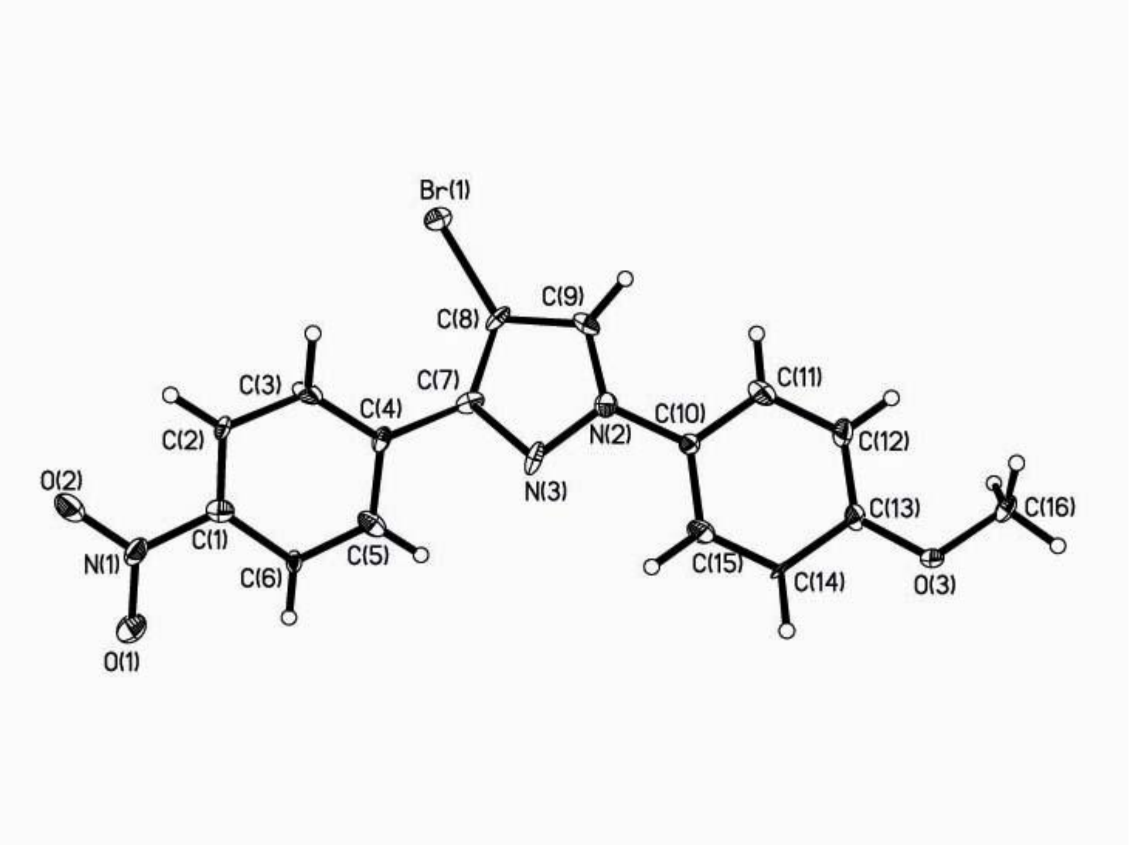

Table 1: Crystal data and structure refinement for mo10609a (pyrazole **3g**).

Identification code	mo10609a (pyrazole **3g**)
Empirical formula	C16 H12 Br N3 O3
Formula weight	374.20
Temperature	173(2) K
Wavelength	0.71073 Å
Crystal system, space group	Monoclinic, *P*2(1)/*n*
Unit cell dimensions	a = 3.9241(19) A alpha = 90 deg.
	b = 22.903(11) A beta = 90.449(7) deg.
	c = 16.432(8) A gamma = 90 deg.
Volume	1476.8(12) Å^3
Z, Calculated density	4, 1.683 Mg/m^3
Absorption coefficient	2.803 mm^−1

F(000)	752
Crystal size	0.20 × 0.15 × 0.04 mm
Theta range for data collection	1.53 to 25.49 deg.
Limiting indices	−4<=h<=4, −23<=k<=27, −19<=l<=19
Reflections collected / unique	6927 / 2743 [R(int) = 0.0809]
Completeness to theta = 25.50	99.6 %
Absorption correction	Semi-empirical from equivalents
Max. and min. transmission	0.8961 and 0.6041
Refinement method	Full-matrix least-squares on F^2
Data / restraints / parameters	2743 / 6 / 209
Goodness-of-fit on F^2	1.341
Final R indices [I>2sigma(I)]	R1 = 0.1567, wR2 = 0.3569
R indices (all data)	R1 = 0.1706, wR2 = 0.3625
Largest diff. peak and hole	2.230 and −4.207 e.Å^−3

Table 2: Atomic coordinates (× 10^4) and equivalent isotropic displacement parameters (Å^2 × 10^3) for mo10609a. U(eq) is defined as one third of the trace of the orthogonalized Uij tensor.

	x	y	z	U(eq)
Br(1)	−1182(5)	8739(1)	5985(1)	27(1)
N(1)	−5930(40)	11705(7)	6423(9)	31(4)
N(2)	2820(40)	8972(6)	8209(9)	24(3)
N(3)	1610(30)	9539(6)	8129(8)	22(3)
O(1)	−6330(50)	12084(7)	6927(9)	51(4)
O(2)	−6660(50)	11787(6)	5704(9)	47(4)
O(3)	9190(30)	8329(5)	11142(7)	30(3)
C(1)	−4320(40)	11145(7)	6625(12)	24(4)
C(2)	−3290(40)	10773(7)	6042(9)	19(3)
C(3)	−1770(50)	10243(8)	6279(11)	26(4)
C(4)	−1270(40)	10102(7)	7097(10)	18(3)
C(5)	−2400(40)	10515(8)	7673(12)	27(4)
C(6)	−3880(40)	11037(7)	7484(9)	21(4)
C(7)	240(40)	9545(7)	7388(10)	21(3)
C(8)	440(40)	8990(8)	7000(9)	23(4)
C(9)	2080(40)	8637(6)	7558(10)	21(4)
C(10)	4460(40)	8805(7)	8957(10)	18(3)
C(11)	5490(40)	8219(8)	9062(11)	24(4)
C(12)	7130(50)	8061(9)	9812(11)	31(4)
C(13)	7700(40)	8459(7)	10420(10)	21(3)
C(14)	6740(50)	9055(8)	10274(11)	35(5)
C(15)	5110(50)	9211(8)	9556(12)	33(4)
C(16)	10280(50)	7745(8)	11273(12)	32(4)

Table 3: Bond lengths [Å] and angles [deg] for mo10609a.

Br(1)-C(8)	1.872(15)
N(1)-O(1)	1.21(2)
N(1)-O(2)	1.23(2)
N(1)-C(1)	1.47(2)
N(2)-C(9)	1.35(2)
N(2)-N(3)	1.39(2)
N(2)-C(10)	1.44(2)
N(3)-C(7)	1.33(2)
O(3)-C(13)	1.35(2)
O(3)-C(16)	1.42(2)
C(1)-C(2)	1.35(2)
C(1)-C(6)	1.44(2)
C(2)-C(3)	1.41(2)
C(2)-H(2)	0.9500
C(3)-C(4)	1.39(2)
C(3)-H(3)	0.9500
C(4)-C(5)	1.41(2)
C(4)-C(7)	1.49(2)
C(5)-C(6)	1.37(2)
C(5)-H(5)	0.9500
C(6)-H(6)	0.9500
C(7)-C(8)	1.42(2)
C(8)-C(9)	1.38(2)
C(9)-H(9)	0.9500
C(10)-C(15)	1.38(2)
C(10)-C(11)	1.41(2)
C(11)-C(12)	1.43(3)
C(11)-H(11)	0.9500
C(12)-C(13)	1.37(3)
C(12)-H(12)	0.9500
C(13)-C(14)	1.44(2)
C(14)-C(15)	1.38(3)
C(14)-H(14)	0.9500
C(15)-H(15)	0.9500
C(16)-H(16A)	0.9800
C(16)-H(16B)	0.9800
C(16)-H(16C)	0.9800
O(1)-N(1)-O(2)	121.2(17)
O(1)-N(1)-C(1)	122.0(15)
O(2)-N(1)-C(1)	116.7(16)

C(9)-N(2)-N(3)	112.8(14)
C(9)-N(2)-C(10)	128.4(14)
N(3)-N(2)-C(10)	118.7(14)
C(7)-N(3)-N(2)	103.4(14)
C(13)-O(3)-C(16)	117.9(14)
C(2)-C(1)-C(6)	123.7(15)
C(2)-C(1)-N(1)	121.5(16)
C(6)-C(1)-N(1)	114.8(14)
C(1)-C(2)-C(3)	118.5(15)
C(1)-C(2)-H(2)	120.7
C(3)-C(2)-H(2)	120.7
C(4)-C(3)-C(2)	121.6(15)
C(4)-C(3)-H(3)	119.2
C(2)-C(3)-H(3)	119.2
C(3)-C(4)-C(5)	116.7(16)
C(3)-C(4)-C(7)	124.2(15)
C(5)-C(4)-C(7)	119.1(15)
C(6)-C(5)-C(4)	124.7(18)
C(6)-C(5)-H(5)	117.6
C(4)-C(5)-H(5)	117.6
C(5)-C(6)-C(1)	114.8(15)
C(5)-C(6)-H(6)	122.6
C(1)-C(6)-H(6)	122.6
N(3)-C(7)-C(8)	112.2(15)
N(3)-C(7)-C(4)	117.6(15)
C(8)-C(7)-C(4)	130.2(15)
C(9)-C(8)-C(7)	104.7(14)
C(9)-C(8)-Br(1)	124.5(13)
C(7)-C(8)-Br(1)	130.8(13)
N(2)-C(9)-C(8)	106.9(14)
N(2)-C(9)-H(9)	126.5
C(8)-C(9)-H(9)	126.5
C(15)-C(10)-C(11)	120.2(16)
C(15)-C(10)-N(2)	120.8(15)
C(11)-C(10)-N(2)	118.9(15)
C(10)-C(11)-C(12)	118.2(16)
C(10)-C(11)-H(11)	120.9
C(12)-C(11)-H(11)	120.9
C(13)-C(12)-C(11)	122.0(17)
C(13)-C(12)-H(12)	119.0
C(11)-C(12)-H(12)	119.0
O(3)-C(13)-C(12)	124.1(16)
O(3)-C(13)-C(14)	117.9(15)
C(12)-C(13)-C(14)	117.9(15)

C(15)-C(14)-C(13)	120.5(16)
C(15)-C(14)-H(14)	119.8
C(13)-C(14)-H(14)	119.8
C(10)-C(15)-C(14)	121.2(17)
C(10)-C(15)-H(15)	119.4
C(14)-C(15)-H(15)	119.4
O(3)-C(16)-H(16A)	109.5
O(3)-C(16)-H(16B)	109.5
H(16A)-C(16)-H(16B)	109.5
O(3)-C(16)-H(16C)	109.5
H(16A)-C(16)-H(16C)	109.5
H(16B)-C(16)-H(16C)	109.5

Symmetry transformations used to generate equivalent atoms:

Table 4: Anisotropic displacement parameters (A^2 x 10^3) for mo10609a. The anisotropic displacement factor exponent takes the form: −2 pi^2 [h^2 a*^2 U11 + ... + 2 h k a* b* U12].

	U11	U22	U33	U23	U13	U12
Br(1)	26(1)	32(1)	22(1)	−5(1)	0(1)	−1(1)
N(1)	40(9)	41(9)	11(8)	7(7)	−17(7)	−10(7)
N(2)	27(8)	19(7)	26(8)	7(6)	4(6)	0(6)
N(3)	21(7)	38(8)	6(6)	9(6)	−3(5)	−3(6)
O(1)	75(12)	40(9)	38(9)	−5(7)	8(8)	10(8)
O(2)	78(12)	18(7)	45(9)	1(6)	16(8)	−4(7)
O(3)	38(7)	28(7)	25(7)	−10(5)	−13(6)	8(6)
C(1)	18(8)	15(8)	39(10)	−4(7)	0(7)	−5(6)
C(2)	29(9)	24(9)	3(7)	6(6)	3(6)	1(7)
C(3)	32(10)	25(9)	23(9)	−6(7)	15(7)	5(7)
C(4)	12(7)	26(8)	16(8)	4(6)	1(6)	−2(6)
C(5)	16(8)	25(9)	41(11)	−3(8)	7(7)	1(7)
C(6)	29(9)	27(9)	6(7)	4(6)	3(6)	13(7)
C(7)	23(8)	25(9)	14(8)	−4(7)	10(7)	−4(7)
C(8)	26(9)	33(9)	8(7)	−5(7)	−11(6)	−2(7)
C(9)	33(9)	3(7)	28(9)	6(6)	18(7)	−4(6)
C(10)	5(5)	18(6)	30(7)	4(6)	−2(5)	4(5)
C(11)	20(9)	30(9)	22(9)	−4(7)	16(7)	1(7)
C(12)	38(10)	37(11)	17(9)	7(8)	−4(8)	13(8)
C(13)	18(8)	28(9)	17(8)	6(7)	−8(6)	0(7)
C(14)	45(11)	34(10)	24(10)	−19(8)	−28(9)	22(9)
C(15)	48(12)	15(8)	37(11)	−5(8)	−4(9)	0(8)
C(16)	37(11)	32(10)	26(10)	−1(8)	−5(8)	7(8)

Table 5: Hydrogen coordinates (x 10^4) and isotropic displacement parameters (A^2 x 10^3) for mo10609a.

	x	y	z	U(eq)
H(2)	−3575	10868	5483	22
H(3)	−1077	9974	5873	31
H(5)	−2095	10423	8233	33
H(6)	−4578	11309	7887	25
H(9)	2593	8234	7495	25
H(11)	5109	7938	8647	28
H(12)	7836	7668	9891	37
H(14)	7220	9345	10672	42
H(15)	4431	9605	9475	40
H(16A)	8291	7497	11373	48
H(16B)	11815	7731	11746	48
H(16C)	11481	7605	10791	48
